# FabR, a regulator of membrane lipid homeostasis, is involved in *Klebsiella pneumoniae* biofilm robustness

**DOI:** 10.1128/mbio.01317-24

**Published:** 2024-09-06

**Authors:** Ibrahima Dramé, Yannick Rossez, Frederic Krzewinski, Nicolas Charbonnel, Laurence Ollivier-Nakusi, Romain Briandet, Etienne Dague, Christiane Forestier, Damien Balestrino

**Affiliations:** 1 Université Clermont Auvergne, CNRS, LMGE, Clermont–Ferrand, France; 2 Université Lille, CNRS, UMR 8576-UGSF-Unité de Glycobiologie Structurale et Fonctionnelle, Lille, France; 3 Université Paris-Saclay, INRAE, AgroParisTech, Micalis Institute, Jouy-en-Josas, France; 4 LAAS-CNRS, CNRS, Univeristé de Toulouse, Toulouse, France; National University of Singapore, Singapore, Singapore

**Keywords:** *Klebsiella pneumoniae*, biofilm robustness, membrane fatty acid, atomic force microscopy

## Abstract

Biofilm is a dynamic structure from which individual bacteria and micro-aggregates are released to subsequently colonize new niches by either detachment or dispersal. Screening of a transposon mutant library identified genes associated with the alteration of *Klebsiella pneumoniae* biofilm including *fabR*, which encodes a transcriptional regulator involved in membrane lipid homeostasis. An isogenic ∆*fabR* mutant formed more biofilm than the wild-type (WT) strain and its trans-complemented strain. The thick and round aggregates observed with ∆*fabR* were resistant to extensive washes, unlike those of the WT strain. Confocal microscopy and BioFlux microfluidic observations showed that *fabR* deletion was associated with biofilm robustness and impaired erosion over time. The genes *fabB* and *yqfA* associated with fatty acid metabolism were significantly overexpressed in the ∆*fabR* strain, in both planktonic and biofilm conditions. Two monounsaturated fatty acids, palmitoleic acid (C16:1) and oleic acid (C18:1), were found in higher proportion in biofilm cells than in planktonic forms, whereas heptadecenoic acid (C17:1) and octadecanoic acid, 11-methoxy (C18:0-OCH3) were found in higher proportion in the planktonic lifestyle. The *fabR* mutation induced variations in the fatty acid composition, with no clear differences in the amounts of saturated fatty acids (SFA) and unsaturated fatty acids for the planktonic lifestyle but lower SFA in the biofilm form. Atomic force microscopy showed that deletion of *fabR* is associated with decreased *K. pneumoniae* cell rigidity in the biofilm lifestyle, as well as a softer, more elastic biofilm with increased cell cohesion compared to the wild-type strain.

**IMPORTANCE:**

*Klebsiella pneumoniae* is an opportunistic pathogen responsible for a wide range of nosocomial infections. The success of this pathogen is due to its high resistance to antibiotics and its ability to form biofilms. The molecular mechanisms involved in biofilm formation have been largely described but the dispersal process that releases individual and aggregate cells from mature biofilm is less well documented while it is associated with the colonization of new environments and thus new threats. Using a multidisciplinary approach, we show that modifications of bacterial membrane fatty acid composition lead to variations in the biofilm robustness, and subsequent bacterial detachment and biofilm erosion over time. These results enhance our understanding of the genetic requirements for biofilm formation in *K. pneumoniae* that affect the time course of biofilm development and the embrittlement step preceding its dispersal that will make it possible to control *K. pneumoniae* infections.

## INTRODUCTION

Bacterial biofilms are complex communities of microorganisms in which cells are embedded in a self-produced matrix of extracellular polymeric substances (EPS) that is protective and adhesive (1). The formation of biofilm involves complex and dynamic events. It is initiated by the bacterial adhesion to a surface, followed by the formation of microcolonies and the progressive production of EPS that give rise to a mature biofilm with a three-dimensional (3D) structure. The EPS matrix, mainly composed of exopolysaccharides, proteins, lipids, and nucleic acids (eDNA and eRNA), provides mechanically stable and complex physicochemical microenvironments (2). In addition, the physical and chemical properties of the biofilm matrix protect against external chemical and biological threats, and degradation caused by mechanical forces (3).

Despite the presence of the matrix that surrounds and cements cells together, a biofilm is a dynamic structure from which individual bacteria and micro-aggregates can colonize new environmental niches, by either detachment or dispersal (4). Detachment, a passive form of cell release, corresponds to biofilm erosion and structural alteration under external forces, such as shear stress. It is greatly influenced by the mechanical characteristics of biofilm, such as elasticity, viscosity, and viscoelasticity (5, 6). The second mechanism, dispersal, is considered an active and highly regulated process that involves the sensing of environmental cues and their transduction through complex regulatory networks to final effectors. Modifications of the biofilm matrix can actively trigger dispersals, such as the production of matrix-degrading enzymes (proteases, desoxyribonucleases, and glycoside hydrolases), disruption of noncovalent interactions between matrix components, and formation of cavities by cell autolysis (4, 7–10). However, the role of the cell membrane fatty acid (FA) composition in biofilm disruption remains unexplored, whereas the ratio of unsaturated fatty acids (UFAs) to saturated fatty acids (SFAs) is the most important parameter affecting membrane fluidity and rigidity in most bacteria (11). Membrane fluidity plays an important role in several physiological cell functions, and the modification of membrane phospholipids is considered to be an adaptative response to withstand external environmental conditions that allow bacteria to limit exchange, conserve energy, and survive within biofilm communities (12, 13). Dubois-Brissonnet et al. (13) showed that the FA content of sessile cells of Gram-positive (*Staphylococcus aureus*, *Listeria monocytogenes*) and Gram-negative (*Pseudomonas aeruginosa*, *Salmonella* Typhimurium) bacteria differs from that of planktonic cells, with SFA content being always higher in biofilm cells than in planktonic cells (13). Similarly, membranes from *S. aureus* biofilm contain more SFAs than membranes in the planktonic stationary phase (14).

By directly affecting membrane fluidity, the FA composition of the membrane could also influence the mechanical properties of the biofilm structure and thus be involved in detachment or dispersal phenomena. In the present work, a library of transposon insertion mutants of *Klebsiella pneumoniae* was screened using experimental models of biofilm formation under static and dynamic conditions. We focused on one of the mutants that showed more robust biofilms over time than the wild-type strain, that is, unable to detach or disperse, in which the gene *fabR*, which encodes a regulator of fatty acid membrane composition, had been disrupted (15). Construction and analysis of an isogenic *K. pneumoniae* mutant deleted for *fabR* and its trans-complement confirmed the role of *fabR* in *K. pneumoniae* biofilm strength. The role of *fabR* was further explored by analyzing and comparing the fatty acid composition of planktonic and biofilm bacterial cells. Finally, atomic force microscopy (AFM) was used to assess the relationship between the nanomechanical properties of *K. pneumoniae* biofilms and their resistance to shear forces, thus clarifying the role of *fabR* at the cellular level and in biofilm development.

## RESULTS

### The *fabR*::TnSC189 mutant formed a structured and robust biofilm over time

A library of 4,032 transposon insertion mutants was created, and the initial screening by Biofilm Ring test selected 259 clones with higher resistance to the mobility of magnetic beads than the *K. pneumoniae* CH1151 strain, suggesting higher robustness of the biofilms. Of the 259 pre-selected mutants, those forming a biofilm capable of withstanding intensive washing in microtiter plates were selected. One of these mutants had inserted the TnSC189 transposon in a gene encoding the fatty acid biosynthesis regulator, *fabR*.

Monitoring biofilm development under dynamic conditions in a BioFlux microfluidic system showed strong erosion and structural alteration over time and from the first hours of formation for the biofilm formed by the CH1151 parental strain. By contrast, the *fabR*::TnSC189 mutant showed robustly structured, rounded aggregates even after 19 hours of incubation. (Fig. S1).

### Deletion of the *fabR* gene leads to increased biofilm formation

**Fig 1:**
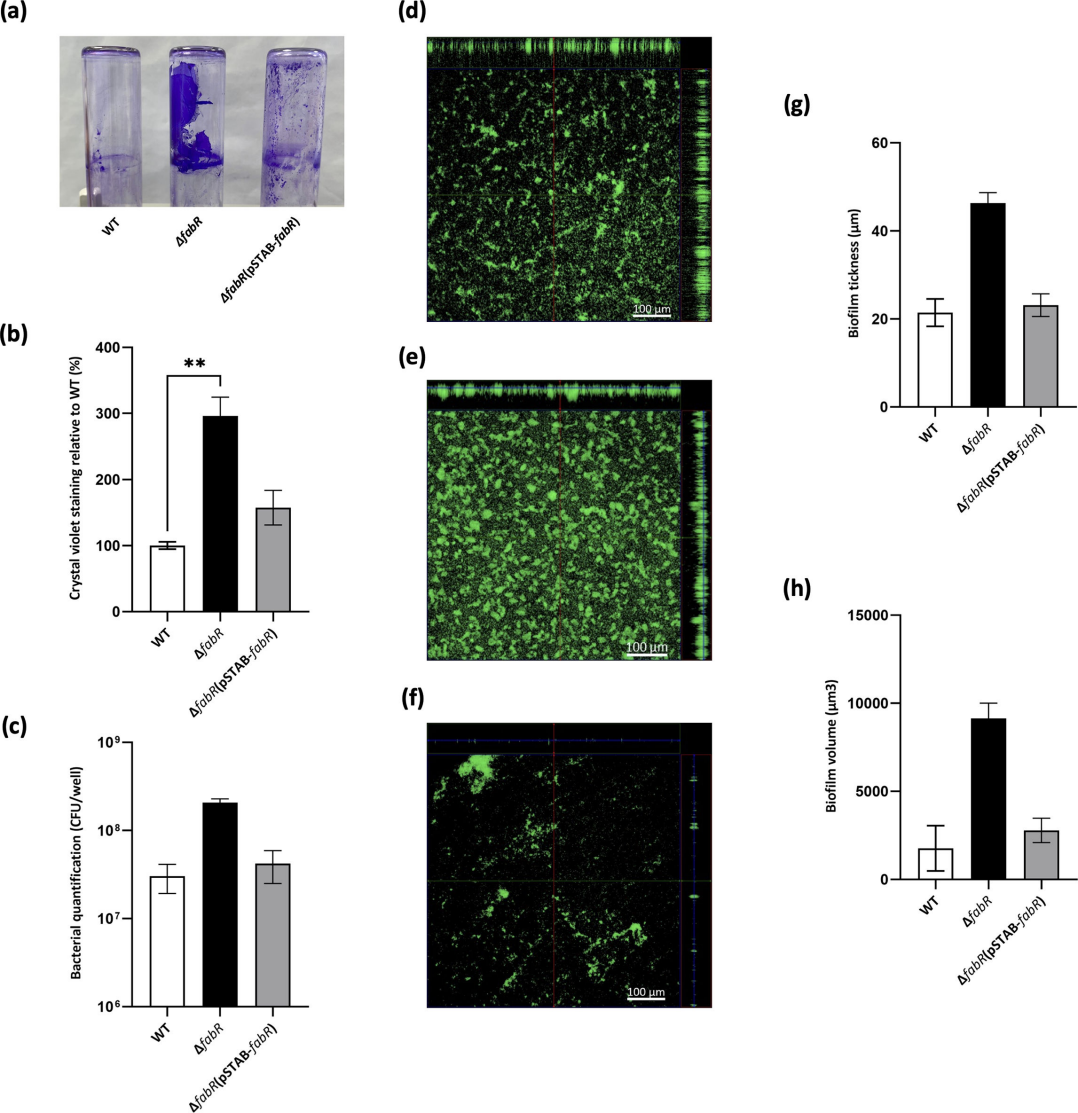
The transcription repressor FabR is related to biofilm formation in *K. pneumoniae*. Biofilms were formed in M63B1-0.4% Glc in glass tubes for 18 hours (**a**), in a 96-well microtiter plate for 5 hours (**b**) and 24-well microtiter plate for 5 hours (**c**), and biomass was then quantified by crystal violet staining or CFU determination as described in Materials and Methods. Biofilms formed by the GFP-expressing WT strains (**d**), *∆fabR* (**e**), and ∆*fabR*(pSTAB-*fabR*) (**f**) in 96-well microtiter plates with coverslip bottom were observed using confocal microscopy after 5 hours of incubation at ×10 magnification. Biovolume (µm^3^) (**g**) and thickness (**h**) of the biofilm formed by each strain were determined using the IMARIS 9.8.2 software package. Data are expressed as means  ±  SEM (*N*  =  3). Statistical analysis: one-way ANOVA with post hoc Dunn’s test (****P ≤ 0.0001; ***P ≤ 0.001; **P ≤ 0.01; *P ≤ 0.05).

An isogenic mutant with *fabR* deleted (∆*fabR*) and its trans-complemented strain ∆*fabR*(pSTAB-*fabR*) were constructed. The growth rates of ∆*fabR* (µ_max_=1.054 ± 0.019) and ∆*fabR*(pSTAB-*fabR*) (µ_max_=1.106 ± 0.072) were not significantly different from those of the WT strain (µ_max_=1.238 ± 0.021) (*P* > 0.05; Kruskal-Wallis with post hoc Dunn’s test). Crystal violet staining of biofilms formed in tubes showed that the ∆*fabR* mutant formed more biofilm on the tube wall than the WT and ∆*fabR*(pSTAB-*fabR*) strains, and the microtiter plate assay indicated that the biofilm biomass of the isogenic mutant was threefold greater than that of the WT (Fig. 1a and b). Quantification of the biofilm biomass by determination of the colony-forming units (CFU) evidenced similar significant differences between the biofilm of ∆*fabR* mutant and those of the WT and trans-complemented strains (Fig. 1c, *P* < 0.05). Unlike the observations on abiotic surfaces, bacterial adhesion to cervical (HeLa) and lung (A549) epithelial cells did not significantly differ between WT and ∆*fabR* strains. Significant differences were only observed between the WT and ∆*fabR*(pSTAB-fabR) for adhesion to A549 cells, and between ∆*fabR* and ∆*fabR*(pSTAB-*fabR*) for adhesion to HeLa cells (Fig. S2).

Confocal laser scanning microscope (CLSM) analysis showed that the biofilm formed by the ∆*fabR* mutant had higher biomass (Fig. 1e) than that of both the WT (Fig. 1d) and ∆*fabR*(pSTAB-*fabR*) strains (Fig. 1f). In addition, the ∆*fabR* mutant biofilm formed structured rounded aggregates that were not observed with the WT and trans-complemented strains. As shown in Fig. 1g, biofilms formed by the ∆*fabR* mutant had a volume average of 9,473 ± 1,499 µm^3^, whereas the biofilm formed by the WT and the ∆*fabR*(pSTAB-*fabR*) strains had averages of 1,762 ± 894 µm^3^ and 2,777 ± 501 µm^3^, respectively (Fig. 1g). Similarly, the ∆*fabR* biofilm had an average thickness of 46.3 ± 3.2 µm versus 21.4 ± 2.4 µm and 23.1 ± 1.8 µm for the WT and the trans-complemented strains, respectively (Fig. 1h).

**Fig 2:**
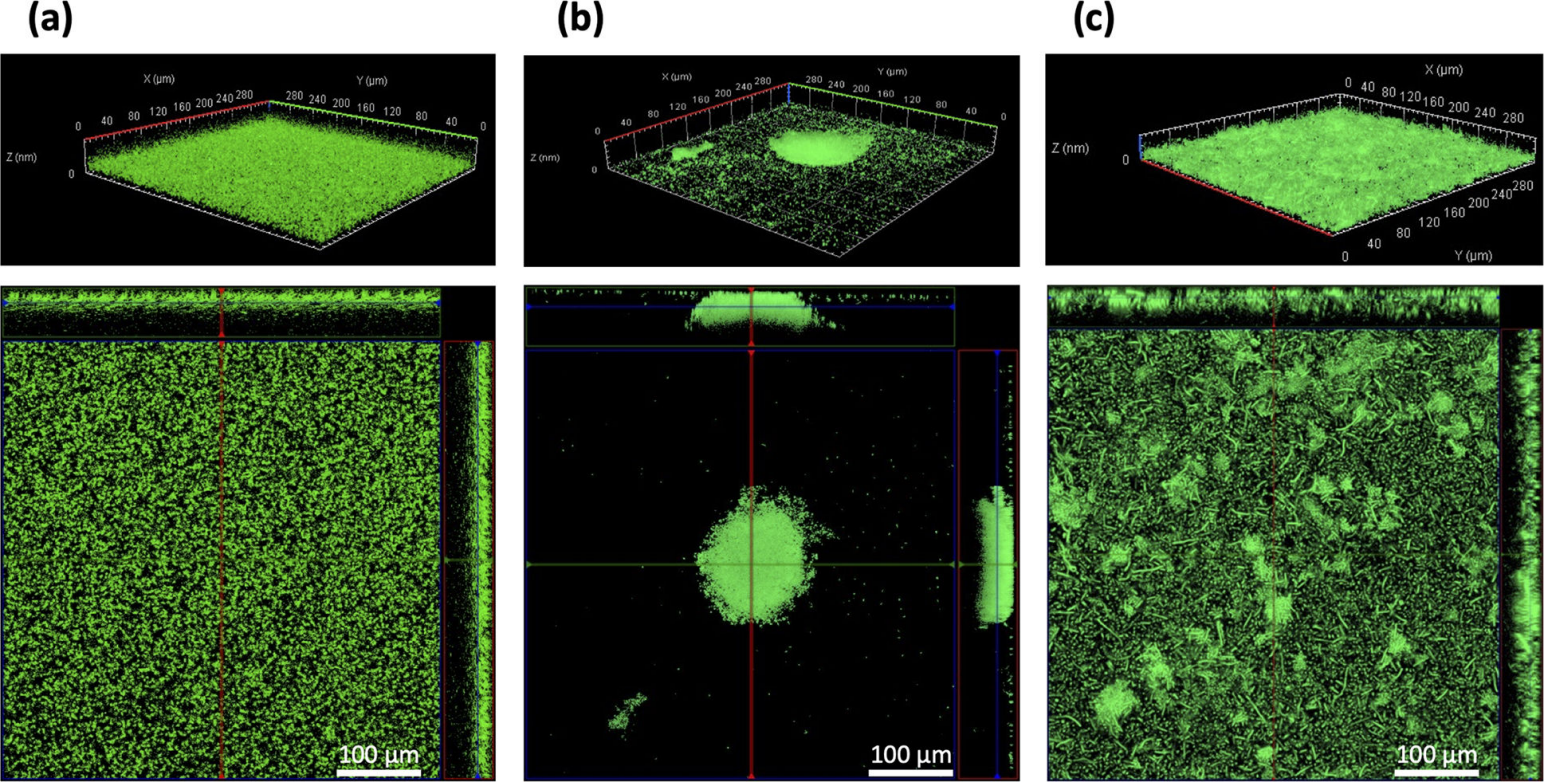
The absence of FabR alters the structure of mature biofilm. The architecture of 24-hour-old biofilms formed by GFP-expressing WT (**a**), *∆fabR* (**b**), and ∆*fabR*(pSTAB-*fabR*) (**c**) strains in 96-well microplates with glass bottom was observed by confocal microscopy. Upper images represent the 3D reconstructions of biofilms, and lower images represent one optical section of the z-stack, with the position indicated by a red line in the upper images. Scale bars correspond to 100 µm. Each image is representative of three independent biological replicates.

When biofilms were maintained in microplate culture for up to 24 hours, the WT strain developed a uniformly covering surface biomass (Fig. 2a), whereas the ∆*fabR* mutant formed thick aggregates with a rounded structure (Fig. 2b). During the extensive washes of the microtiter plates, the biofilm formed by the WT and trans-complemented strains became detached, suggesting a biofilm fragility not observed with the ∆*fabR* mutant. Biofilm formed by the ∆*fabR*(pSTAB-*fabR*) strain had a similar appearance to that seen on the WT, with several small aggregates and elongated bacteria (Fig. 2c).

### Role of *fabR* in the alteration of *K. pneumoniae* biofilm structure under dynamic conditions

**Fig 3:**
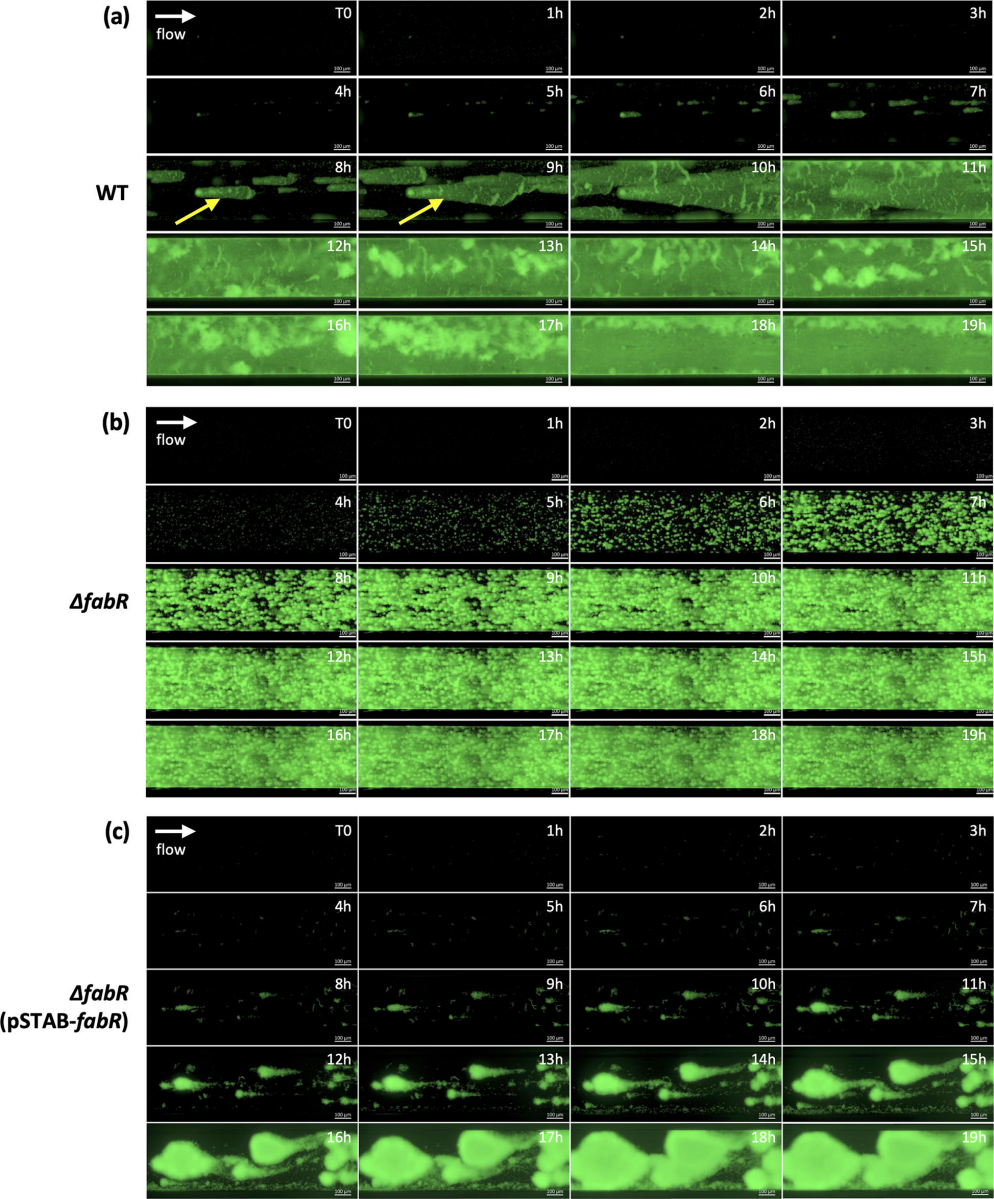
The absence of FabR leads to a stable biofilm without aggregate detachment and shear-erosion over time. The kinetics of biofilm formation by the GFP-expressing WT (**a**), *∆fabR* (**b**), and ∆*fabR*(pSTAB-*fabR*) (**c**) strains was followed in the BioFlux microfluidic system at 37°C under a shear force of 0.5 dyn/cm² using epifluorescence microscope (Axio observer 7, Zeiss) at a magnification of 20×. Images were acquired in real time at T = 0 hour and then every hour, and each image represents one time point. The yellow arrows indicate the comet-like structures formed in the WT strain. The white arrows indicate the flow direction. Each image sequence is representative of three independent biological replicates.

Figure 3a presents the kinetics of biofilm formation by the WT strain in the Bioflux microfluidic model. Initially, the adherent bacteria formed structured microcolonies, which grew and spread in the direction of flow, forming comet-like structures and eventually covering the surface within 12 hours of incubation (Fig. 3a). As they developed, some biofilm aggregates detached to form flocs that were carried away by the flow. By contrast, the ∆*fabR* mutant formed microcolonies with a rounded shape that grew and were not altered by shear forces over time (Fig. 3b; Fig. S3). Neither comet-like structures nor aggregate detachment and erosion were observed during biofilm development by the ∆*fabR* strain. Biofilm formed by the ∆*fabR*(pSTAB-*fabR*) trans-complemented strain was different from those formed by the WT and the ∆*fabR* strains had large around aggregates that did not cover the surface (Fig. 3c).

**Fig 4:**
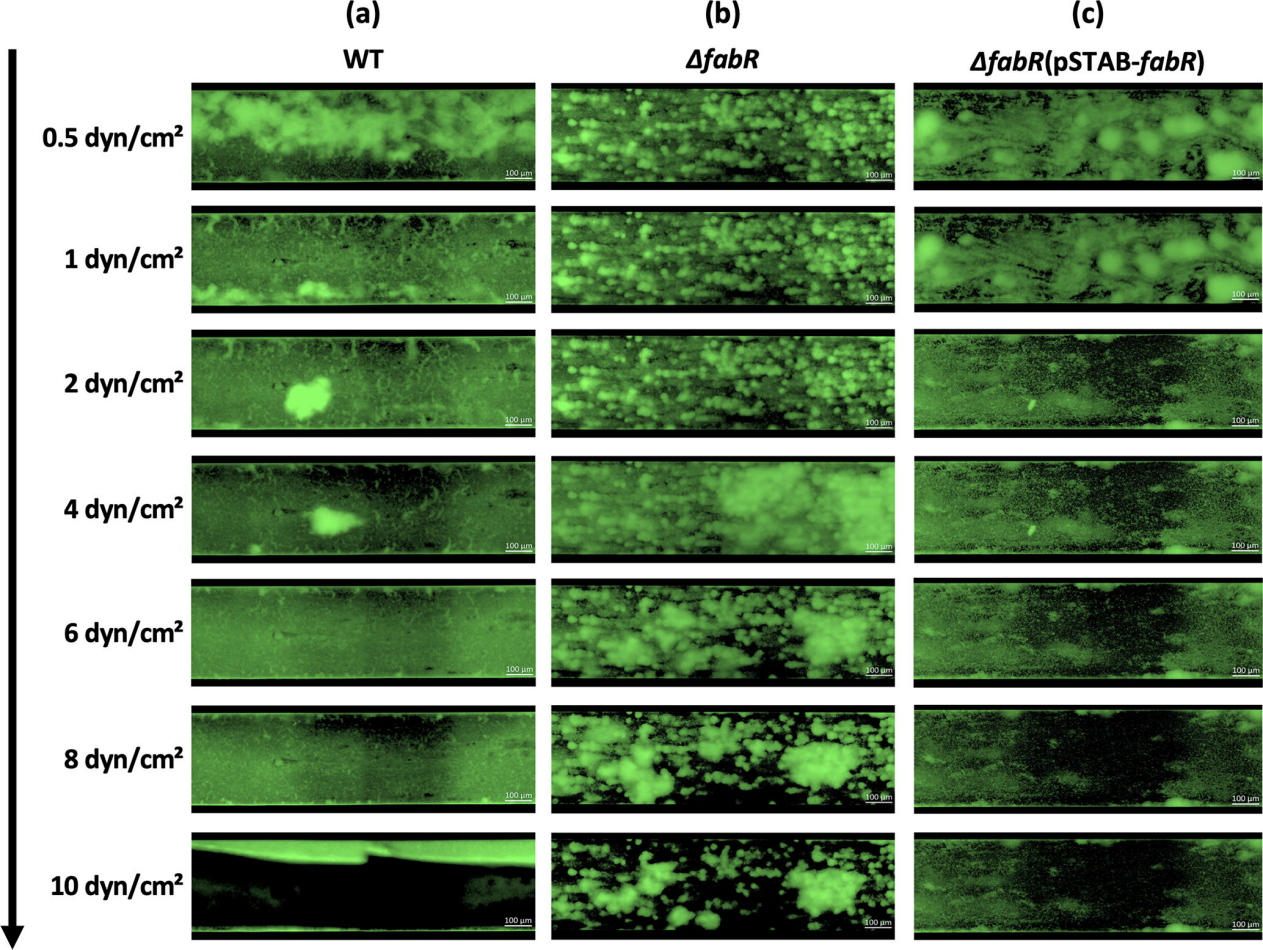
The absence of FabR leads to the formation of a robust biofilm more resistant to shear forces than the WT strain. The behavior of *K. pneumoniae* biofilm under increasing shear forces was analyzed in the BioFlux microfluidic system using an epifluorescence microscope (Axio observer 7, Zeiss) at a magnification of 20×. Twenty-four-hour-old biofilms formed by the WT (**a**), ∆*fabR* (**b**), and ∆*fabR*(pSTAB-*fabR*) (**c**) strains under shear forces of 0.5 dyn/cm² were subjected to a gradually increasing shear force from 0.5 to 10 dyn/cm^2^. Images were acquired after each increase in shear force. Each image sequence is representative of three independent biological replicates.

The robustness of the biofilm formed after 19 hours of incubation was analyzed by gradually increasing the shear force from 0.5 to 10 dyn/cm^2^. Significant erosion of the WT biofilm was observed at 1 dyn/cm² (Fig. 4a) while the ∆*fabR* biofilm resisted up to an increase of 4 dyn/cm² (Fig. 4b). Inside the biofilm formed by the ∆*fabR* strain, some aggregates resisted to shear force up to 10 dyn/cm^2^. As observed with the WT strain, a biofilm of ∆*fabR*(pSTAB-*fabR*) became detached when the shear force increased to 1 dyn/cm² (Fig. 4c).

### *fabR regulates fabA* and *fabB* genes in sessile bacteria

**Fig 5:**
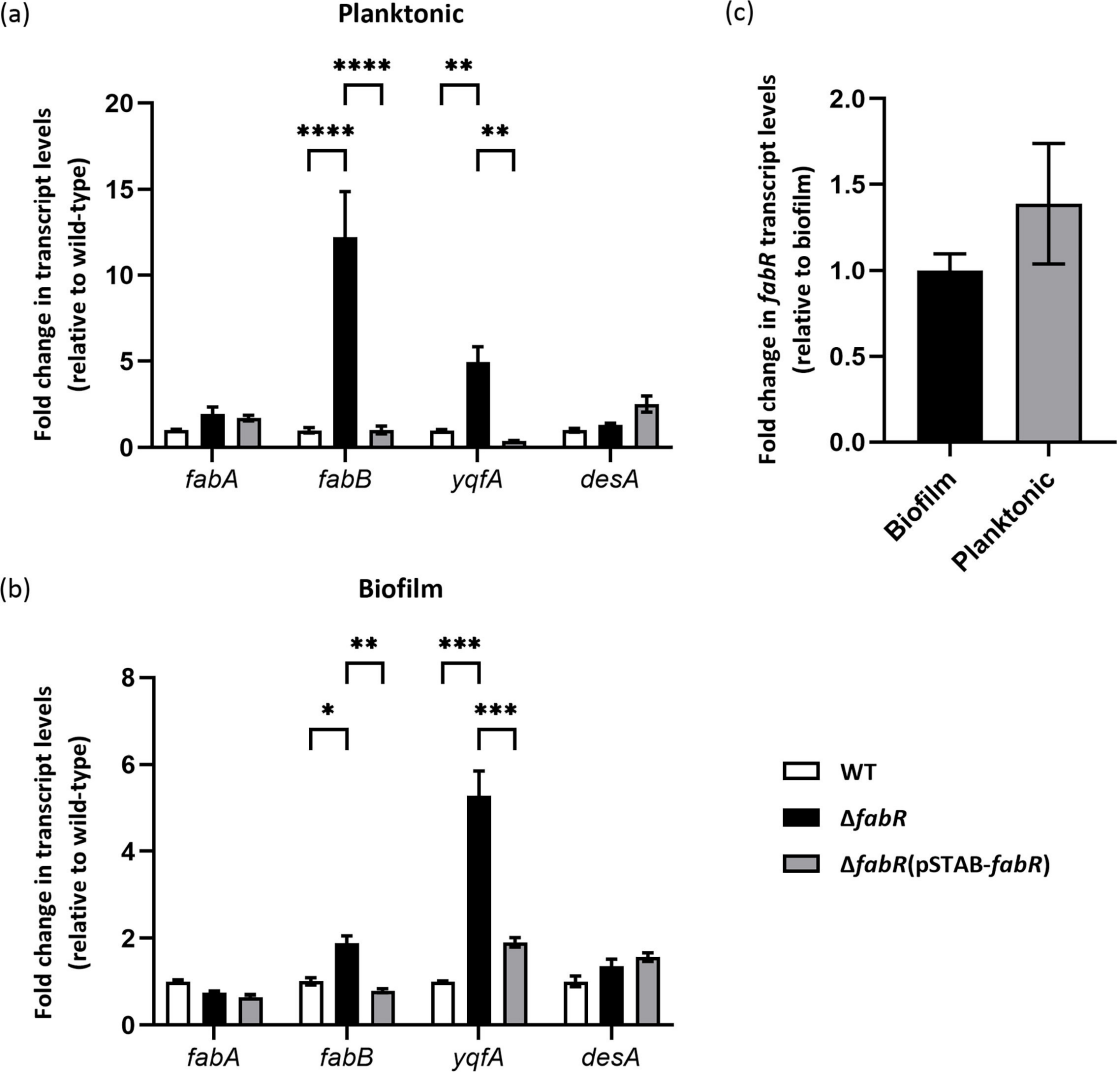
FabR regulates the expression of *fabB* and *yqfA* genes in planktonic and biofilm cells. Differential expressions of the genes *fabA*, *fabB*, *yqfA,* and *desA* involved in fatty acid metabolism in *K. pneumoniae* WT, ∆*fabR,* and ∆*fabR*(pSTAB-*fabR*) strains were determined by RT-qPCR. RNA isolated from (**a**) planktonic culture and (**b**) 24-hour-old biofilm formed by the indicated strains were used, and the fold change in transcript abundance was determined relative to the transcript abundance values of the WT strain. (**c**) Analysis of *fabR* gene expression by RT-qPCR in planktonic bacteria compared to that in bacteria grown in biofilm. The fabR expression in biofilm is used as the reference condition. The expression data were normalized using both *rpoD* and *proC* reference genes. Data are expressed as means  ±  SEM (*N*  =  3). Statistical analysis: two-way ANOVA with post hoc Tukey test (***P ≤ 0.001; **P ≤ 0.01; *P ≤ 0.05).

RT-qPCR was used to quantitatively determine the expression of the *fabA*, *fabB,* and *yqfA* genes, previously described as part of the FabR regulon, and the expression of a putative fatty acid desaturase DesA_FADS-like (*desA*) encoded in the *K. pneumoniae* genome. No significant difference was observed in the transcript levels of *fabA* and the DesA_FADS-like encoding gene *desA* between the WT, ∆*fabR,* and ∆*fabR*(pSTAB-*fabR*) strains in planktonic (Fig. 5a) and sessile bacteria (Fig. 5b), indicating that neither the deletion of *fabR* nor its overexpression (in the trans-complemented strain) affected the expression of these genes. However, we observed significant overexpression of the *fabB* and *yqfA* genes in the ∆*fabR* strain compared with the WT and ∆*fabR*(pSTAB-*fabR*) strains in both planktonic and biofilm conditions (Fig. 5). Although the induction of expression of *yqfA* was similar between the planktonic and biofilm conditions, the induction of *fabB* expression was more pronounced in the planktonic condition (12.2-fold change between WT and ∆*fabR*) than in the biofilm (1.9-fold change between WT and ∆*fabR*) (Fig. 5). Analysis of *fabR* expression showed no significant difference in transcript levels in WT between the planktonic and biofilm growth conditions (Fig. 5c). The *fabR* gene was strongly overexpressed in the transcomplemented strain compared to the WT strain, with 170-fold overexpression in biofilm cells and 230-fold overexpression in planktonic cells (data not shown), which could explain the lack of the restoration of wild-type phenotype in some models.

### *fabR* and the bacterial membrane fatty acid composition in planktonic and biofilm cultures

**Fig 6:**
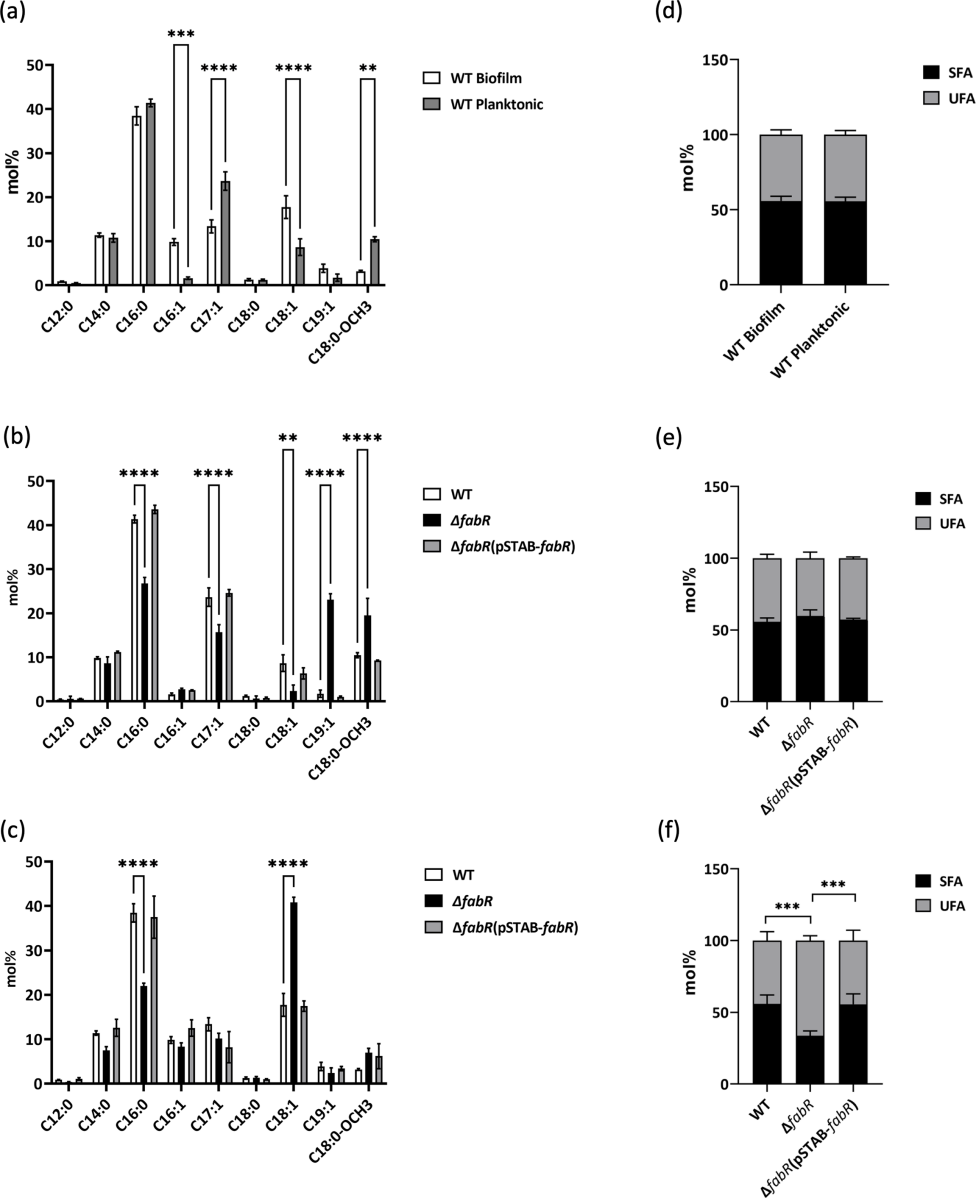
Identification and quantification of the total fatty acid composition in planktonic and biofilm cultures were conducted using GC-MS and GC-flame ionization detector. Fatty acid results are presented in mol% for both biofilm and planktonic cultures in the WT strain (**a and d**). The fatty acid content was also analyzed in WT, ∆*fabR*, and ∆*fabR*(pSTAB-*fabR*) planktonic cultures (**b and e**) and biofilm cultures (**c and f**). The amount of SFA and UFA in mol% was determined for each corresponding condition (d, e, and f). The results correspond to at least three or four biologically independent samples. Statistical significances were determined by post hoc Tukey test after a two-way analysis of variance (****P ≤ 0.0001; ***P ≤ 0.001; **P ≤ 0.01; *P ≤ 0.05).

Extraction and analysis of the fatty acid composition of *K. pneumoniae* WT grown in biofilm and planktonic lifestyles revealed that two monounsaturated fatty acids, palmitoleic acid (C16:1) and oleic acid (C18:1), were higher in biofilm cells compared to planktonic forms. Conversely, heptadecenoic acid (C17:1) and octadecanoic acid, 11-methoxy (C18:0-OCH3) were higher in the planktonic lifestyle (Fig. 6a). The amounts of saturated and unsaturated fatty acids (SFA and UFA, respectively) did not differ between the two lifestyles (Fig. 6d). We then evaluated the effect of the *fabR* mutation on the planktonic lifestyle by comparing the fatty acid content of the WT and the ∆*fabR*(pSTAB-*fabR*) strains (Fig. 6b). Palmitic acids (C16:0), C17:1, and C18:1 content were significantly lower in Δ*fabR* than in the WT, whereas nonadecanoic acids (C19:1) and C18:0-OCH3 were seen in greater amounts in Δ*fabR*. Identification of these unusual fatty acids was performed by gas chromatography-mass spectrometry (GC-MS) as shown in Fig. S4. Across all conditions, no clear differences in the sum of SFA and UFA were found for the planktonic lifestyle (Fig. 6e). The same experiment was conducted for the biofilm lifestyle (Fig. 6c). Only C18:1 showed an increase in Δ*fabR*, while C16:0 was found at a significantly lower percentage. This result was confirmed by absolute quantification, showing a significant increase in C18:1 for Δ*fabR* in biofilm (Fig. S5).

### *fabR* and the nanomechanical properties of *K. pneumoniae* cells

**Fig 7:**
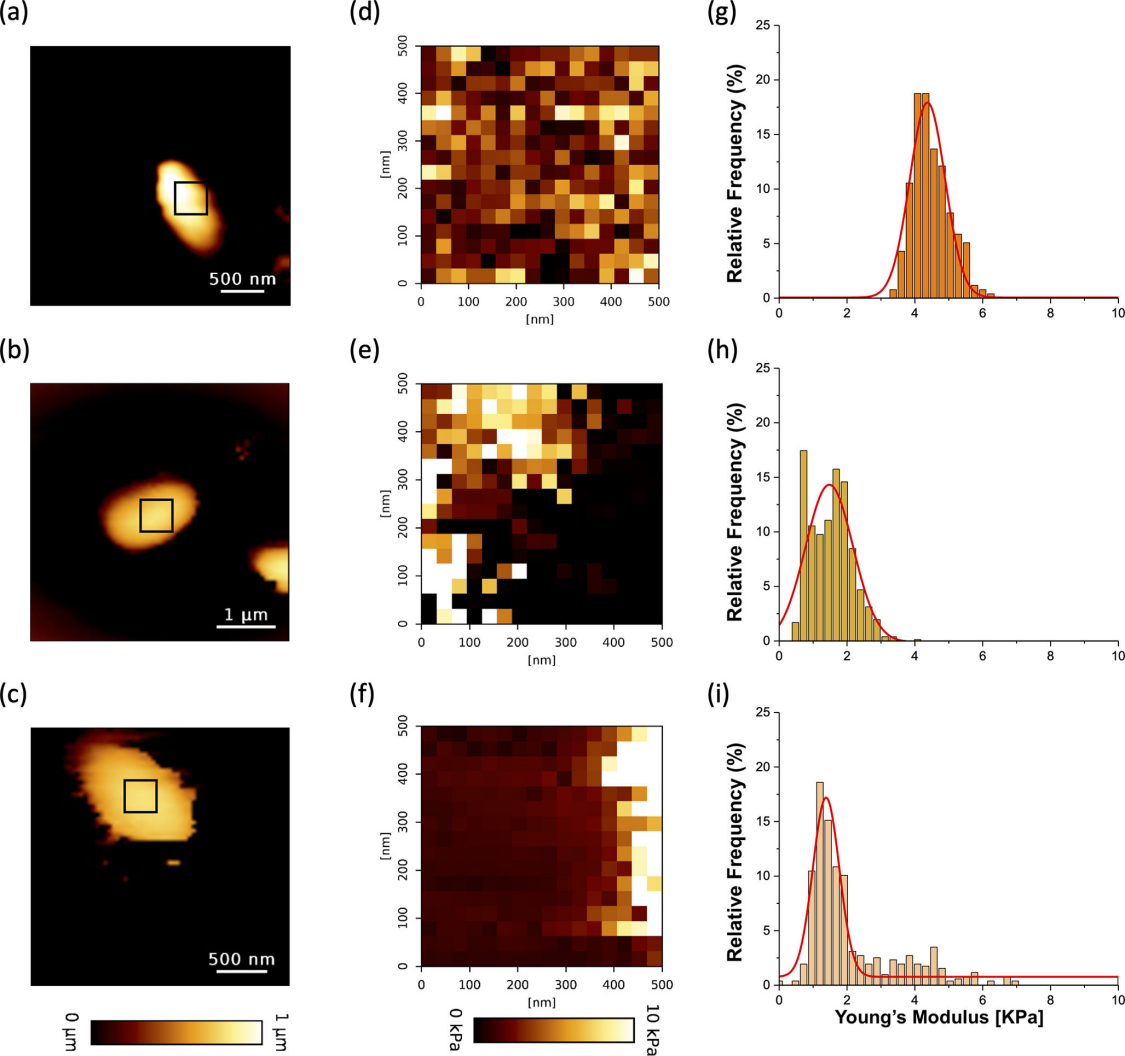
Imaging and nanomechanical properties of *K. pneumoniae* single cell. Height image of WT (**a**), ∆*fabR* (**b**), and ∆*fabR*(pSTAB-*fabR*) (**c**) individual cell interactions between the AFM colloidal probe (silica microsphere) and single planktonic cells were probed on areas of 500 × 500 nm on top of each strain. Elasticity maps (**d–f**) and statistical distribution of the Young’s modulus (**g–i**) were determined for each strain (respectively WT (**d, g**), ∆*fabR* (**e, h**), and ∆*fabR*(pSTAB-*fabR*) (**f, i**). Force curves were recorded on four cells from three independent cultures (a total of 256 force curves per cell corresponding to 16 × 16 pixels).

Atomic force microscopy (AFM) was used in the quantitative imaging mode to characterize the morphology and the nano mechanical properties of the bacteria. In this mode, bacteria are kept alive, in liquid while the tip is scanned in the z-direction according to a matrix of points. On each point of the matrix, a force curve is recorded from which mechanical properties and morphology are extracted which allows us to reconstruct point-by-point height (Fig. 7a through c) or elasticity maps (Fig. 7d through f). Quantitative imaging (QI) mode provided height images showing the morphology (rod-shaped bacteria) of single cells from WT (Fig. 7a), ∆*fabR* (Fig. 7b), and ∆*fabR*(pSTAB-*fabR*) (Fig. 7c). ∆*fabR* cells showed dark contrast close to 0 kPa compared to the WT (Fig. 7d and e) and trans-complemented (Fig. 7f) strain maps, which have elastic modulus values close to 5–10 kPa. The distributions of Young’s modulus values for each strain [WT, ∆*fabR,* and ∆*fabR*(pSTAB-*fabR*)] are presented as histograms in Fig. 7g through i. Young’s modulus values for ∆*fabR* cells were in the average of 1.29 ± 0.51 kPa for 99.2% of extend force curves (Fig. 7h) versus 4.37 ± 0.52 kPa in WT cells (99.2% of curves) (Fig. 7g). The ∆*fabR* cells were therefore three times softer than those of WT. Young’s modulus values determined for the ∆*fabR*(pSTAB-*fabR*) cells (Fig. 7i) were not significantly different (1.48 ± 0.39 kPa for 81.6% of curves) from those of the ∆*fabR* cells. All these results indicate that *fabR* expression is associated with an increase in *K. pneumoniae* cell rigidity in biofilm lifestyle.

### *fabR* and the nanomechanical properties of *K. pneumoniae* biofilms

**Fig 8:**
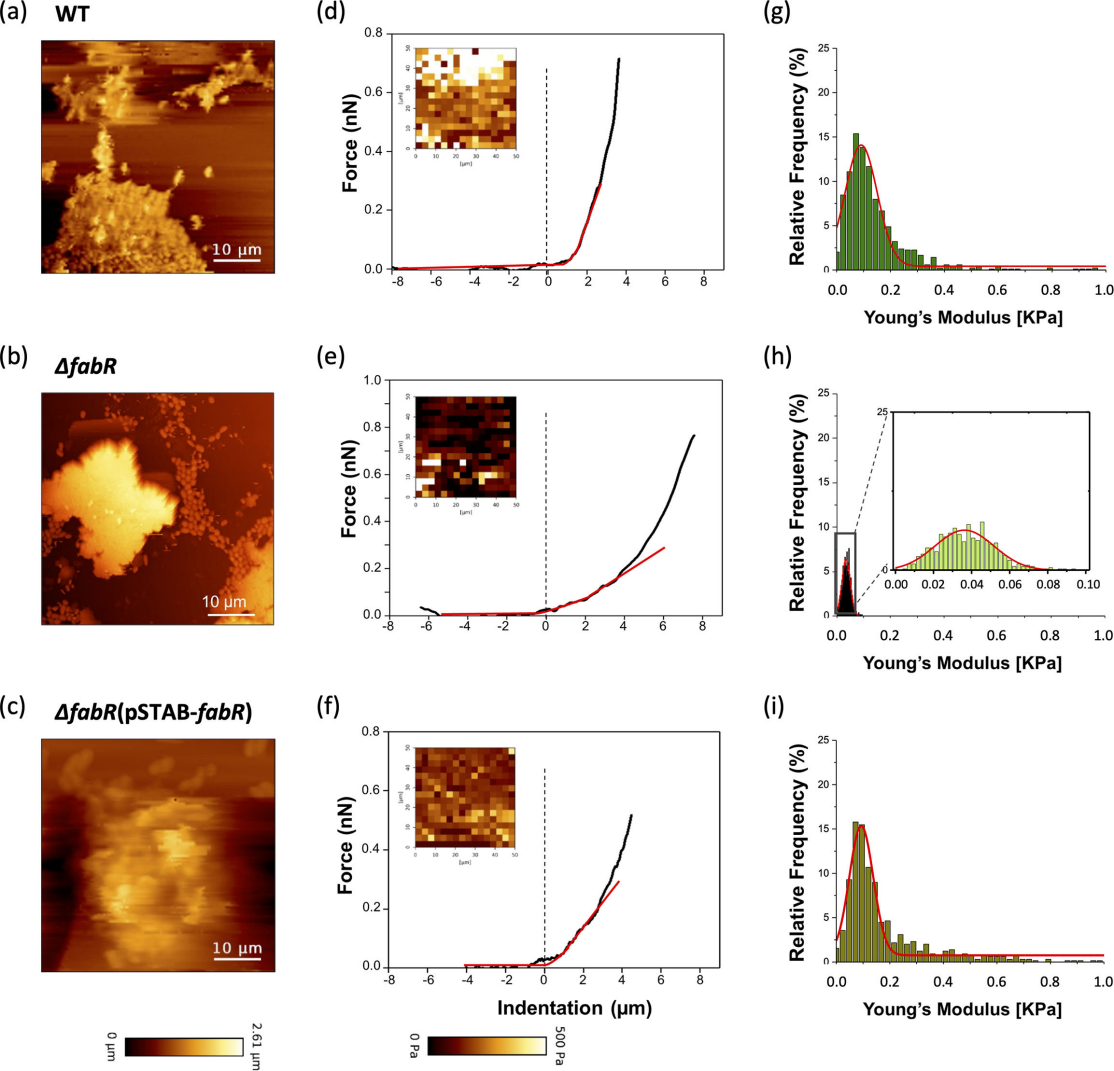
Imaging and nanomechanical properties of *K. pneumoniae* biofilms. Height images of 5-hour-old biofilms of WT (**a**), ∆*fabR* (**b**), and ∆*fabR*(pSTAB-*fabR*) (**c**) were recorded in QI mode. Measurement of the interaction between AFM colloidal probe and area of 50 µm × 50 µm on formed biofilms. (**d–f**) Elasticity maps and representative force-indentation curves were obtained on each biofilm (black line), fitted with the Hertz model (red line). (**g–i**) Statistical distribution of Young’s modulus determined for each strain. Force curves were recorded on three areas of biofilm and from three independent cultures (a total of 256 force curves per measure corresponding to 16 × 16 pixels).

Height images generated by QI mode showed the topography of the 5-hour-old biofilm surface and interface of WT (Fig. 8a), ∆*fabR* (Fig. 8b), and ∆*fabR*(pSTAB-*fabR*) (Fig. 8c). Other QI maps made on aggregates showed clearly visible bacteria embedded in their matrix of EPS (Fig. S6). To determine the nanomechanical properties of the biofilms formed by the wild type and its mutants, force spectroscopy experiments were performed with spherical AFM probes. The elastic modulus values of biofilms from WT, ∆*fabR,* and ∆*fabR*(pSTAB-*fabR*) obtained by recording a total of 2.034 force-distance curves per strains showed heterogeneous contrast and elastic modulus values close to 0.5 kPa for the WT strain (Fig. 8d) and a dark contrast close to 0 kPa for the ∆*fabR* strain (Fig. 8e). The maps from the ∆*fabR*(pSTAB-*fabR*) strain (Fig. 8f) were similar to those of the WT strain. Representative force-indentation curves obtained from each biofilm are presented in Fig. 8d through f. The highest indentation was observed in ∆*fabR* biofilm (4 µm of indention depth for an applied force of 2 nN), whereas the WT and ∆*fabR*(pSTAB-*fabR*) biofilms both had indentations of 2 µm for the same applied force (Fig. 8e), meaning that ∆*fabR* biofilm was softer than WT or ∆*fabR*(pSTAB-*fabR*) biofilms. The quantitative analysis of Young’s modulus distribution is presented as histograms (Fig. 8g through i). For ∆*fabR* biofilm, Young’s modulus values were in the average of 0.04 ± 0.02 kPa (Fig. 8h). The Young’s modulus determined in WT and ∆*fabR*(pSTAB-*fabR*) were in the average of 0.12 ± 0.06 kPa and 0.09 ± 0.04 kPa, respectively, confirming that the biofilm of ∆*fabR* mutant was softer and more elastic than those formed by the WT and ∆*fabR*(pSTAB-*fabR*) strains.

### *fabR* and cell cohesiveness in *K. pneumoniae* biofilms

**Fig 9:**
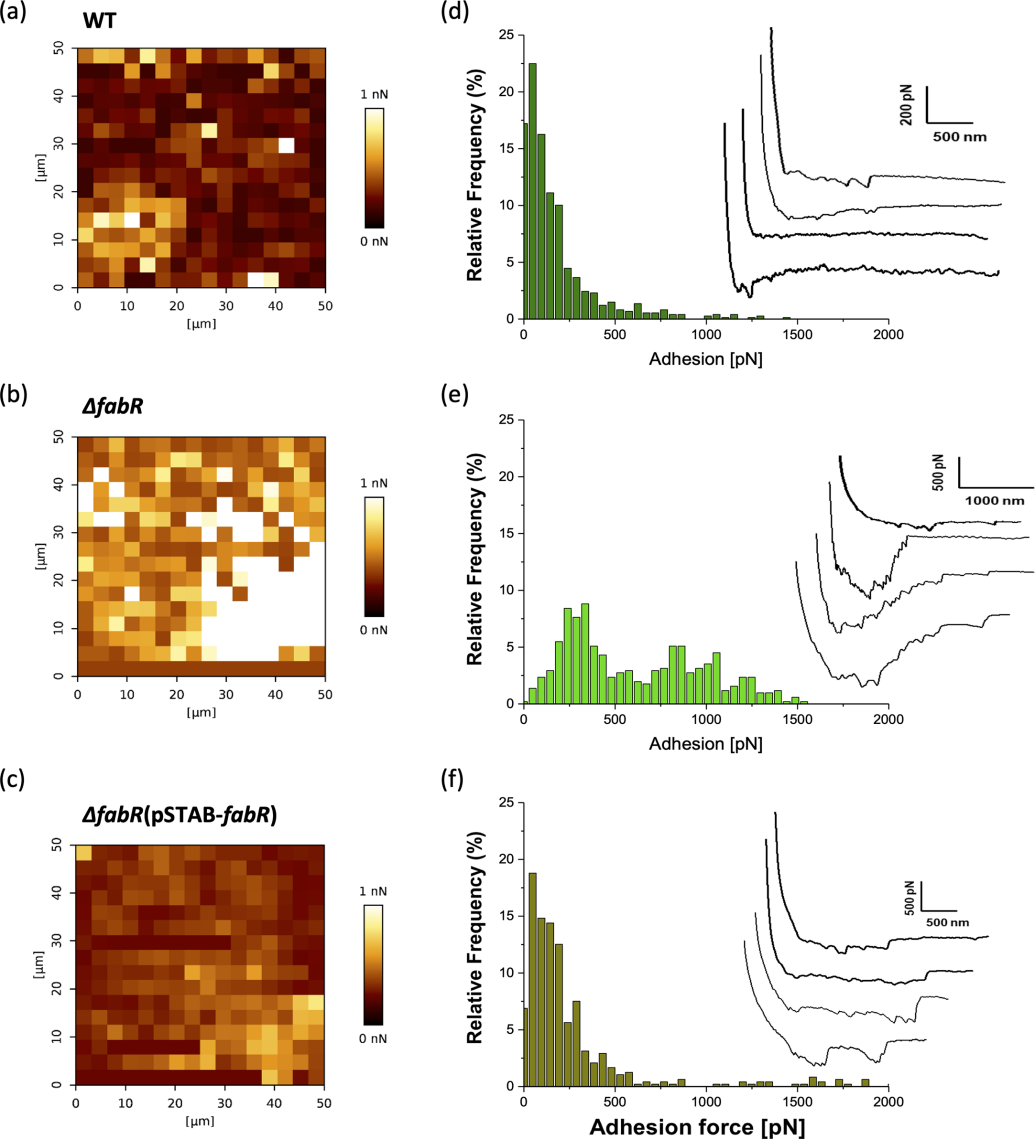
Homotypic interactions between individual cells and 5-hour-old biofilms of *K. pneumoniae*. Adhesion force maps (**a–c**), adhesion force histograms, and typical force-distance curves (**d–f**) were obtained by recording force curves on 50 µm × 50 µm biofilm surface in WT (**d**), ∆*fabR* (**e**), and ∆*fabR*(pSTAB-*fabR*) (**f**) strains. All force curves were recorded on biofilm from three independent cultures and with 16 × 16 pixels per condition corresponding to 256 force curves.

Quantification of the homotypic interactions between individual cells immobilized on AFM colloidal probes and a 5-hour-old biofilm interface showed maps that had adhesion force values (Fig. 9a through c) and typical force-distance curves (Fig. 9d through f). The adhesion forces obtained for the ∆*fabR* mutant varied from 250 to 1,250 pN (*n* = 768 curves) and were an average of 893 ± 29 pN for 91.1% of the force distance curves analyzed (Fig. 9e). By contrast, the results obtained with the WT (*n* = 713 curves) and ∆*fabR*(pSTAB-*fabR*) (*n* = 741 curves) strains were similar and ranged from 50 to 250 pN, in average of 140 ± 32 pN and 159 ± 70 pN, respectively. In addition, the typical force curves had signatures with larger peaks in the ∆*fabR* mutant than in the WT strain, evidence of a strong interaction between biofilm cells. All these results support the involvement of *fabR* in the decrease in cell cohesiveness in *K. pneumoniae* biofilm.

## DISCUSSION

In this study, we show that the regulator FabR, which is involved in UFA biosynthesis, is associated with biofilm robustness in *K. pneumoniae*. In *Escherichia coli*, this transcriptional repressor is part of the machinery that adjusts the UFA:SFA ratio present in the membrane in response to the composition of the cellular pool of long-chain acyl-thioesters (16). In response to high concentration of unsaturated fatty acid thioesters (of either acyl carrier protein or CoA), FabR has an enhanced affinity for binding to and thus repressing the promoters of *fabA* and *fabB*, two genes involved in UFA biosynthesis such as palmitoleic acid and oleic acid (17). FabA catalyzes the formation of cis-3-decenoyl-ACP, and FabB elongates cis-3-decenoyl-ACP to cis-5-dodecenoyl-ACP, which enters the standard fatty acid synthesis cycle and becomes elongated to the 16- and 18-carbon UFAs (12, 17–19). In *P. aeruginosa,* FabR (also known as DesT) not only modulates the expression of the *fabAB* operon but also controls the expression of the *desCB* operon that encodes an oxygen-dependent acyl-CoA Δ9–desaturase that converts SFA-CoA (16:0- or 18:0-CoA) to Δ9-UFA-CoA (16:1Δ9- or 18:1Δ9-CoA) (18).

At the individual bacteria scale, modifications of fatty acyl chains of membrane lipids are essential and play a crucial role in membrane homeostasis, survival, and growth, allowing passive permeability of hydrophobic compounds and modulating the function of membrane-associated proteins, fluidity, and viscosity (20). Bacterial cells can modify their membrane fatty acid composition in response to environmental cues such as temperature, osmolarity, pH, presence of toxic compounds, and exogenous fatty acids (13, 18, 21–23). The transition from the planktonic to the biofilm states, which can be considered as an adaptive stress response, could also be associated with a change in membrane fatty acid composition. By comparing the fatty acid composition of the membrane of biofilm cells with that of their free-growing counterparts, we observed that the UFAs:SFAs ratio was similar between planktonic and sessile states in *K. pneumoniae*. However, a greater amount of palmitoleic acid (C16:1) and oleic acid (C18:1) and a reduced amount of C17:1 and C18:0-OCH3, a methyl-branched fatty acid (MBFA), were observed in the cells from biofilm than in planktonic forms (Fig. 6a and d). These differences appear to be independent of *fabR* expression, as no difference in its transcript level was observed between the two growth conditions (Fig. 5c). In contrast to our findings, Dubois-Brissonnet et al. (13) showed that the membrane FA profiles of some Gram-negative and Gram-positive bacteria (*S. aureus, L. monocytogenes, P. aeruginosa,* and *S*. Typhimurium) present significantly higher proportions of SFAs in the biofilm state compared to the proportions observed into planktonic cells. However, Y. Wang et al. (24) recently showed a FA shift in *L. monocytogenes* from planktonic to biofilm state, with higher concentrations of UFAs and straight FA in biofilm cells than in planktonic cells. Results of studies of *P. aeruginosa* are conflicting, with some reporting a lower concentration of linear SFAs and fatty acids with ≥16 carbons in biofilm cells than in planktonic cultures, and this composition is likely to confer more fluid biophysical properties to cell membranes (25), and others observing a drastic decrease in uneven-chain phospholipids and an accumulation of long-chain lipids in biofilm, suggesting greater lipid stability in the bilayer and reduced membrane fluidity (26). Of note, MBFAs are commonly found in bacteria where they tend to diminish lipid condensation, reduce bilayer thickness, and decrease chain ordering, leading to an increase in membrane fluidity. This effect is attributed to the formation of kinks at the branching points (27); hence, the reduced presence of C18:0-OCH3 in *K. pneumoniae* biofilm may significantly influence its fluidity.

The absence of FabR in *K. pneumoniae* resulted in a robust biofilm, without aggregate detachment and erosion over time observed with wild-type strain. These results differ from those obtained by Hermans et al. (28) with *S*. Typhimurium, where a *fabR* knock-out mutant was impaired in its ability to form biofilm. This defect was attributed to direct FabR-dependent regulation of FabB since a *fabB*-overexpressing strain partly mimics the biofilm defect of the *fabR* mutant. The authors hypothesized that the enhanced expression of *fabB* in the ∆*fabR* mutant lead to increased levels of free UFAs, which could act as biofilm-dispersing molecules. However, the addition of exogenous UFA (cis-5-dodecenoic acid, cis-9-hexadecenoic acid, and cis-11-hexadecenoic acid) to the growing biofilm had no impact, thereby ruling out this hypothesis (28). The fatty acid composition of *S*. Typhimurium also includes cyclopropane fatty acids, which were not detected in our study. In addition, hydroxylated C14:0 was inconsistently detected, making up less than 1% of all fatty acids, and was therefore not considered further. By contrast, in *S*. Typhimurium this type of fatty acid represents about 9% of the total fatty acids. Furthermore, neither C18:0-OCH3 nor C19:1 was detected in *S*. Typhimurium (29). The implications of these differences in fatty acid composition between the two bacterial species are unknown but could directly influence membrane properties and the subsequent fatty acid synthesis by FabA and FabB. In our study, lipid content analysis of sessile *K. pneumoniae* cells in the absence of FabR revealed an increase in C18:1 and an overall decrease in SFA (Fig. 6c and f). These results are consistent with the known role of FabR in UFA synthesis via FabB. When the bacteria were grown in the planktonic form, the deletion of *fabR* led to no variation of the UFAs:SFAs ratio between the wild type and the ∆*fabR* mutant (Fig. 6b and e). The elevated expression of *fabB* in the ∆*fabR* mutant likely accounts for the observed increase in longer fatty acid chains in planktonic cells (30). It is noteworthy that free UFAs, known for their role in biofilm dispersal, typically consist of small UFAs of C10 to C16 carbon length (31). Consequently, overexpression of FabB, which leads to the production of longer fatty acid chains, is likely to deplete the pool of smaller UFAs required for FabB activity.

The differences in fatty acids observed between the two lifestyles of ∆*fabR* mutant suggest a complex regulation of the enzymes involved in the biosynthesis of monoenoic fatty acids in *K. pneumoniae*.

To unravel how FabR impinges on *K. pneumoniae* biofilm formation, we performed AFM experiments to determine the rigidity/elasticity of both individual bacteria and biofilm. We propose that, in addition to modulating fatty acid composition and biofilm structure, FabR is involved in nanomechanical properties and thus influences biofilm resistance/erosion under dynamic conditions since high levels of UFA tend to increase membrane fluidity, while straight-chain SFAs cause a loss of fluidity (32). In our study, when *fabR* is deleted, the cells lose their rigidity (Fig. 7), meaning that the maintenance of cell rigidity is probably due to the adjustment of the UFA:SFA ratio in the WT strain.

At the biofilm level, while the deletion of *fabR* increased the ratio of UFA (C18:1) in the membrane of biofilm cells, a decrease in cell rigidity was observed, leading to a robust, softer, and elastic biofilm able to resist high shear forces. These observations suggest that FabR, by modulating lipid composition, reduces *K. pneumoniae* biofilm strength and makes it stiffer with a compact structure that makes the biofilm more easily detachable under dynamic conditions. In addition, AFM images showed that ∆*fabR* biofilm cells are covered with a higher matrix content than those of WT (Fig. S6), which probably contributes to the biofilm’s softness and elasticity, and thus to resistance under shear force over time.

There has been no documented report directly linking biofilm structure, fatty acid composition, nanomechanical properties, and flow forces. Certain studies have shown that pili are involved in the elasticity of *Lactococcus lactis* biofilm (33) without evidencing, however, the impact of the elasticity properties of biofilms on their resistance to shear forces, despite the well-known role of pili in aiding cells to withstand shear flow (34). In our study, we also posited the potential role of FabR in cell adhesion within biofilms. As shown in Fig. 9, the high adhesion level of the ∆*fabR* mutant was probably due to the biofilm elasticity and resulted in stronger cohesion between biofilm cells than in wild-type cells which had low adhesion forces.

In summary, we described the role of the transcriptional regulator FabR in modulating the UFA:SFA ratio in *K. pneumoniae* cell membranes and thus its influence on the global structure of the biofilm, with alteration of the biofilm structure over time being correlated with biofilm stiffness. Altogether, our results provide an understanding of the molecular mechanism induced by FabR in the biofilm embrittlement that precedes the dispersion process *in K. pneumoniae*.

## MATERIALS AND METHODS

### Bacterial strains, plasmids and growth conditions

Bacterial strains and plasmids used in this study are given in Table 1. The ampicillin-sensitive *K. pneumoniae* CH1157 ∆*shv::aadA7* was taken as the wild-type strain (35). *K. pneumoniae* and *E*. coli strains were grown at 37°C with shaking at 200 rpm in M63B1 minimal medium containing 0.4% (wt/vol) of D-glucose (M63B1-0.4% Glc) and lysogeny broth (LB), respectively. Bacterial density was estimated by optical density measurement at 620 nm (OD_620_) as we previously determined that one DO unit is equivalent to ∼10^9^ CFU/mL for a liquid culture of *K. pneumoniae*. For determination of the bacterial growth rate, bacteria were cultivated in M63B1-0.4% Glc in 96-well microtiter plates at 37°C, and OD_620_ was measured every 30 min using Spark Cyto (TECAN). The µ_max_ was obtained on the basis of the slope calculated in accordance with the exponential growth curve between 2.5 and 5 hours of culture.

**TABLE 1:** Strains and plasmids used in this study^*a*^

Strain or plasmid	Description	Antibiotic resistances	Source or reference
Strains			
*K. pneumoniae*			
WT1151	CH1151 (CH1034 clinical isolate ∆*shv::aadA7-gfpmut3*)	Sp	(36)
*fabR*::TnSC189	CH1151 *fabR*::TnSC189	Sp, Km	This work
WT	CH1157 ∆*shv::aadA7*	Sp	(35)
WT-*gfp*	Mini-Tn7T-Km-*gfpmut3* inserted into *att*Tn7 sites of the wild-type strain	Sp, Km	This work
∆*fabR*	Isogenic ∆*fabR* deletion	Sp	This work
∆*fabR-gfp*	Mini-Tn7T-Km-*gfpmut3* inserted into *att*Tn7 site of ∆*fabR*	Sp, Km	This work
*E. coli*			
TOP10	F− *mcrA* Δ(*mrr-hsd*RMS-*mcr*BC) Φ80*lac*ZΔM15 Δ*lac*X74 *rec*A1 *ara*D139 Δ(*araleu*) 7697 *gal*U *gal*K *rps*L (StrR) *end*A1 *nup*G		Invitrogen
MFD λpir	MG1655 RP4-2-Tc::[∆Mu1::*aac* (3)*IV-∆aphA-nic35*-Mu2::*zeo*]∆*dapA*::(*erm-pir*) ∆*recA*	Ap, Zeo, Erm	(37)
Plasmids			
pSC189	RP4-dependent origin of conjugation (*oriT*), Π-dependent origin of replication (*ori* R6K), mariner-based transposon TnSC189 and C9 transposase	Ap, Km	(38)
pSTAB	pZE derivative plasmid, with the flm toxin-antitoxin system from F plasmid, Ap^R^	Ap	Gift from JM Ghigo
pSTAB-*fabR*	pSTAB plasmid with *fabR* gene under the control of its native promoter	Ap	This work
pKD4	Template for Km^R^ cassette	Km	(39)
pKOBEG119	λ red recombinase expression vector	Tet	(40)
pCP20	Flippase helper plasmid	Ap	(39)
pTNS3	Helper plasmid, harboring the *tnsABCD* gene required forTn7 transposition function. R6K *ori*, *oriT*	Ap	(41)
pUC18R6KT-mini-Tn7T-Km	pUC18-based delivery plasmid for mini-Tn7-Km. R6K *ori*, *oriT*	Ap, Km	(41)
pUC18R6KT-mini-Tn7T-Km-*gfp*	pUC18-based delivery plasmid for mini-Tn7-Km-*gfp* harboring GFPmut3 encoding gene. R6K *ori*, *oriT*	Ap, Km	(35)

^*a*^ Abbreviations: Ap: ampicillin 100 µg/mL; Sp: spectinomycin 50 µg/mL; Km: kanamycin 50 µg/mL; Zeo: zeocine 50 µg/mL; Erm: erythromycin 200 µg/mL; Tet: tetracyclin 35 µg/mL.

### Construction of a mutant library, screening and sequencing

A mutant library was constructed using the *E. coli* MFD*pir*(pSC189) to deliver a TnSC189 kanamycin-resistant *mariner*-based transposon into the *K. pneumoniae* CH1151 strain (Table 1). A total of 4,032 Km^R^ transconjugants were selected and screened by two successive methods: (i) the Biofilm Ring test (Biofilm Control, Saint-Beauzire, France) as described by Dos Santos Goncalves et al. (42) and (ii) a confocal microscopy approach. Mutants forming a biofilm that entrapped more beads after 5 hours of incubation than that of the WT in the Biofilm Ring test were selected for the second screening by confocal microscopy as described below. Those exhibiting more biomass than the wild-type strain after two extensive washes were selected. Next-generation Illumina sequencing (Helixio, Saint-Beauzire, France) was used to locate TnSC189 insertion sites in the mutants of interest.

### Strain constructions

PCRs were performed using Phusion High-Fidelity DNA polymerase (New England BioLabs) according to the manufacturer’s recommendations. All the primers are shown in Table S1. The *K. pneumoniae* ∆*fabR* isogenic mutant was constructed by the replacement of *fabR* by a kanamycin resistance encoding gene that was secondarily excised (39, 40). The GFP encoding gene was introduced into the chromosome of the *K. pneumoniae* strains using a mobilizable mini-Tn7 base vector by triparental mating involving *E. coli* MFD*pir* harboring the plasmid pUC18R6KT-mini-Tn7T-Km-*gfp*, *E. coli* MFD*pir*(pTNS3) and the recipient strain (41).

For trans-complementation, a fragment containing the *fabR* gene and its promoter was amplified from *K. pneumoniae* CH1157 genomic DNA and cloned into the PCR-amplified pSTAB plasmid using HiFi cloning kit (New England BioLabs). The resulting recombinant plasmid pSTAB-*fabR* was then transformed by electroporation into the ∆*fabR* and ∆*fabR-gfp* strains, giving rise to the trans-complemented ∆*fabR*(pSTAB-*fabR*) and ∆*fabR-gfp*(pSTAB-*fabR*) strains, respectively (Table 1). The empty pSTAB plasmid was introduced into the WT and ∆*fabR* strains, and all analyses were performed with strains harboring pSTAB plasmid.

### Biofilm formation

Pre-cultures and biofilm experiments were performed in M63B1-0.4% Glc at 37°C. Biofilms were formed on the wall of glass tubes for 18 hours with agitation at 200 rpm. After careful removal of bacterial suspensions, biofilms were washed twice with PBS, fixed with 80%, and stained with crystal violet solution (0.1%, wt/vol). After two washes with H_2_O, crystal violet was solubilized in acetic acid 33% (wt/vol) solution, and absorbance was measured at 595 nm. In 96-well and 24-well microtiter plates (polystyrene flat bottom, Falcon), biofilms were formed using, respectively, 2.10^5^ CFU and 2.10^6^ CFU of overnight cultures. After incubation for 5 hours with agitation at 200 rpm, quantification of biofilms was performed by crystal violet staining as described above and by determination of the number of colony-forming units (CFU) after scraping the well surface and plating of serial dilutions. For CLSM observation, biofilms of GFP-expressing *K. pneumoniae* strains were formed in black 96-well plates with coverslip bottom (Greiner Bio-One) for 5 and 24 hours at 37°C. After being thoroughly rinsed twice with PBS and fixed with paraformaldehyde (3%), the biofilms were observed at 10× magnification (ZEISS LSM 800 with Airyscan) and images were processed using Zen 2 (blue edition) software. The IMARIS 9.8.2 software package (Bitplane, Belfast, United Kingdom) was then used to generate the 3D projections of biofilms and to determine the average biovolume and thickness.

The biofilm development in the microfluidic system using the BioFlux 200 system (Fluxion Biosciences, South San Francisco, USA) device is described in supplemental materials and methods.

### Preparation of biofilm and planktonic extracts for gene expression and biochemical analyses

Pyrex biofilm microfermentors containing a removable Pyrex glass spatula (V.S.N., Paris, France) were used to grow biofilms under continuous flow conditions as described by Ghigo (43). After 24  hours of continuous culture at 90  mL/h, the total biofilm biomass (Fig. S7) was recovered by vortexing in 15 mL of physiological water. For planktonic extracts, bacteria were cultivated in 50 mL of M63B1-0.4% Glc for 24 hours at 37°C under agitation at 200 rpm. Each biofilm and exponential culture condition was prepared in biological triplicate.

### RNA extraction and reverse transcription-quantitative PCR assay

Planktonic bacteria and biofilms recovered from the glass spatula of the microfermentors were used to perform total RNA extraction according to the method described by Toledo-Arana et al. (44). Reverse transcription was performed with 500 ng of total RNA and qPCRs were carried out using primers listed in Table S1. The protocols for RNA extraction and RT-qPCR are detailed in the supplemental materials and methods.

### Biochemical analysis of the fatty acid composition in planktonic and biofilm cultures

The bacterial suspensions from biofilms and planktonic cultures were centrifuged at 4,500 × *g* for 10 min at 4°C and the bacterial pellets collected in 5 mL milli-Q water were lyophilized for biochemical analysis. The initial step of the lipid extraction process was performed by the Bligh and Dyer method. This analysis used a BPX70 column and followed established methods as detailed in Tao et al. (22), allowing separations of fatty acid methyl esters (FAMEs) of a given chain length that differ by degree of unsaturation, positions of double bonds, and functional groups (e.g., methoxylated fatty acids). For absolute quantification, we added 12.5 µg of pentadecanoic acid (C15:0) to 7.5 mg of dried biofilm prior to lipid extraction. To identify individual fatty acids, a standard containing 37 FAMEs (Sigma Aldrich, Trace CERT) was used and further validated through gas chromatography coupled with mass spectrometry. This process involved injecting 1 µL of the sample in splitless mode on a Solgel 1 MS capillary column (30 m × 0.25 mm × 0.25 µm) with a temperature gradient of 120 to 230°C at a rate of 3°C/min, followed by an increase to 270°C at 10°C/min. The compounds were detected at 70 eV using an HP-7820 gas chromatograph coupled to a 5977B single quad (Agilent Technologies, Santa Clara, CA, USA) in full scan mode across the mass range from 45 to 500 Da. All cultures were performed in biological triplicates.

### AFM imaging experiments

AFM images were assessed in PBS at room temperature using the Quantitative Imaging mode (QI) available on the Nanowizard III AFM (JPK-Bruker Instruments, Berlin, Germany) (45). Planktonic cells were washed in PBS and immobilized on 2% of polyethylenimine (PEI)-coated glass slides as described by Francius et al. (46) and Formosa et al. (47). For biofilm observation, biofilms formed on a coverslip for 5 hours at 37°C with shaking were rinsed with PBS and placed on a glass slide. MLCT cantilevers C (Bruker, USA, nominal spring constant of ⁓0.01 N/m) were used with the parameters defined by Drame et al. (33). All images acquired were analyzed with data processing software from JPK-Bruker instruments (version 6.1.198).

### Force spectroscopy measurements

The nanomechanical properties of *K. pneumoniae* planktonic cells and biofilms were determined by force spectroscopy experiments using a Nanowizard III AFM (JPK-Bruker, Berlin, Germany). AFM colloidal probes were used and prepared according to the protocol described by Dramé et al. (48). Forces of 2 nN were applied with z length between 3 and 15 µm and approach-retract speed between 10 and 30 µm/s. Indentation curves and Young’s modulus were determined as described in Drame et al. (33).

The adhesion forces between individual bacteria and biofilm interfaces were measured with AFM colloidal probes as described in Drame et al. (33) and analyzed with data processing software from JPK-Bruker Instruments (version 6.1.198).

### Statistics

Statistical analyses were performed with GraphPad Prism 9 (GraphPad Software; La Jolla, CA, USA) and IBM Spss Statistic 25. Differences were considered significant at *P* < 0.05 (*), 0.01 (**), and 0.001 (***).

## SUPPLEMENTAL MATERIAL

Figure S1 mbio.01317-24-s0001.pdf The insertion of the TnSC189 kanamycin-resistant mariner-based transposon into the *fabR* gene of the *K. pneumoniae* CH1151 strain led to a stable biofilm without aggregates detachment and shear-erosion over time.

Figure S2 mbio.01317-24-s0002.pdf The deletion of the transcription repressor FabR does not impact the adhesion of *K. pneumoniae* in eucaryotic cell monolayer.

Figure S3 mbio.01317-24-s0003.pdf The deletion of *fabR* is associated to the formation of microcolonies with a rounded structure-shape that grew and were not altered by shear forces over time.

Figure S4 mbio.01317-24-s0004.pdf Details of the gas chromatography data obtained.

Figure S5 mbio.01317-24-s0005.pdf Identification and quantification of the total fatty acid composition in biofilm cultures were conducted using GC-MS and GC-FID.

Figure S6 mbio.01317-24-s0006.pdf AFM QI images of *K. pneumoniae* biofilms showed bacteria embedded in their extracellular matrix of EPS.

Figure S7 mbio.01317-24-s0007.pdf The deletion of *fabR* led to an increase biofilm formation.

Supplemental text mbio.01317-24-s0008.pdf Supplemental materials and methods.

TABLE S1 mbio.01317-24-s0009.pdf Primers used in this study.

ASM does not own the copyrights to Supplemental Material that may be linked to, or accessed through, an article. The authors have granted ASM a non-exclusive, world-wide license to publish the Supplemental Material files. Please contact the corresponding author directly for reuse.
