# Supplementary material for: FabR, a regulator of membrane lipid homeostasis, is involved in Klebsiella pneumoniae biofilm robustness
Source: mBio. 2024 Sep 6;15(10):e01317-24. doi: 10.1128/mbio.01317-24 (PMC11481535; doi:10.1128/mbio.01317-24)
Supplement: Figure S1 — The insertion of the TnSC189 kanamycin-resistant mariner-based transposon into the fabR gene of the K. pneumoniae CH1151 strain led to a stable biofilm without aggregates detachment and shear-erosion over time. [file mbio.01317-24-s0001.pdf]

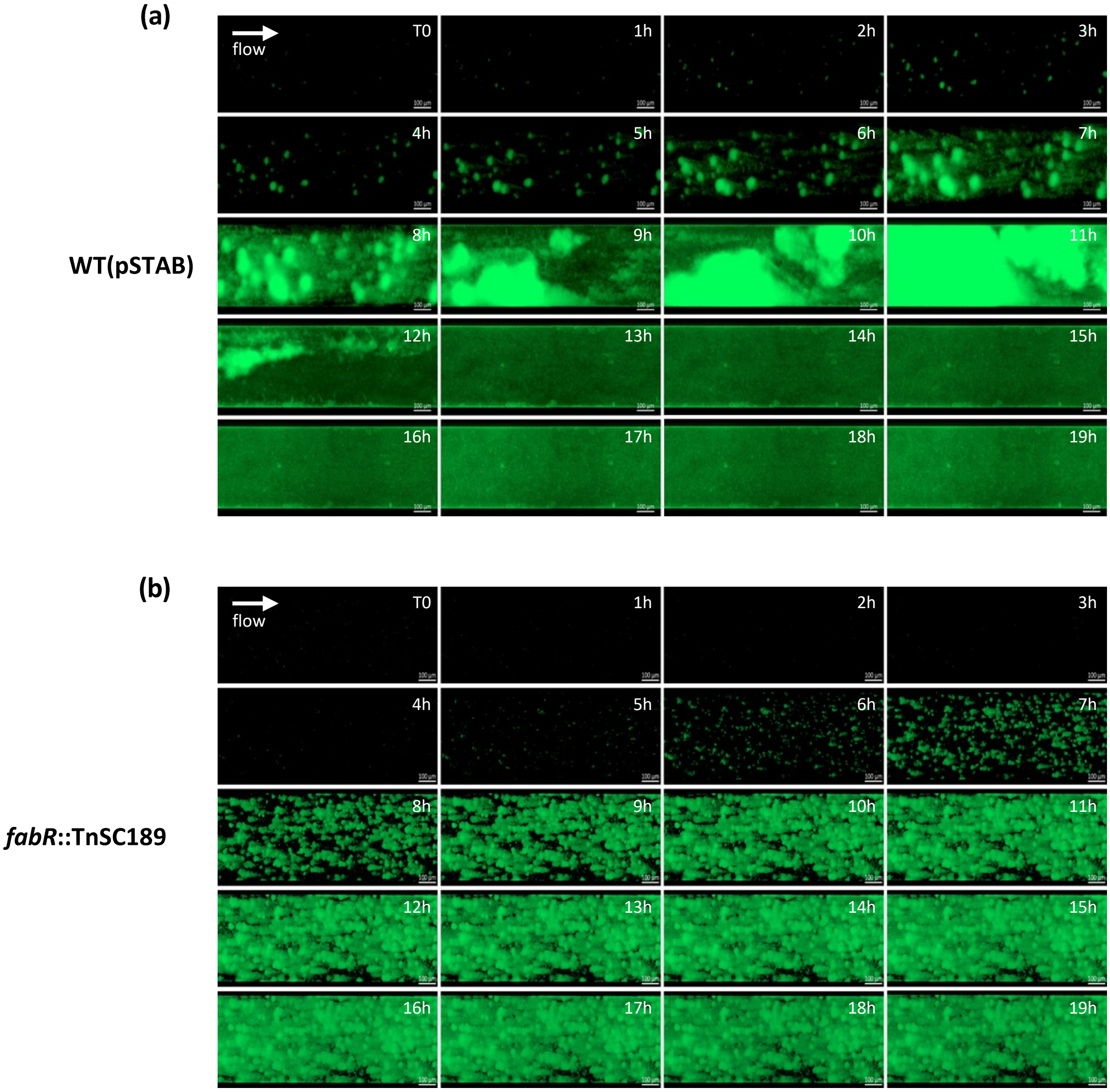

Fig. S1. The insertion of the TnSC189 kanamycin-resistant mariner-based transposon into the *fabR* gene of the *K. pneumoniae* CH1151 strain led to a stable biofilm without aggregates detachment and shear-erosion over time. The kinetic of biofilm formation by the GFP-expressing WT1151 (a) and *fabR*::TnSC189 (b) strains was followed in the BioFlux^{TM} microfluidic system at 37°C under a shear force of 0.5 dyn/cm^2 using epifluorescence microscope (Axio observer 7, Zeiss) at the magnification of 20 ×. Images were acquired in real time at T = 0 hour and then every hour, and each image represents one time point. The white arrows indicate the flow direction
