# Supplementary material for: FabR, a regulator of membrane lipid homeostasis, is involved in Klebsiella pneumoniae biofilm robustness
Source: mBio. 2024 Sep 6;15(10):e01317-24. doi: 10.1128/mbio.01317-24 (PMC11481535; doi:10.1128/mbio.01317-24)
Supplement: Figure S2 — The deletion of the transcription repressor FabR does not impact the adhesion of K. pneumoniae in eucaryotic cell monolayer. [file mbio.01317-24-s0002.pdf]

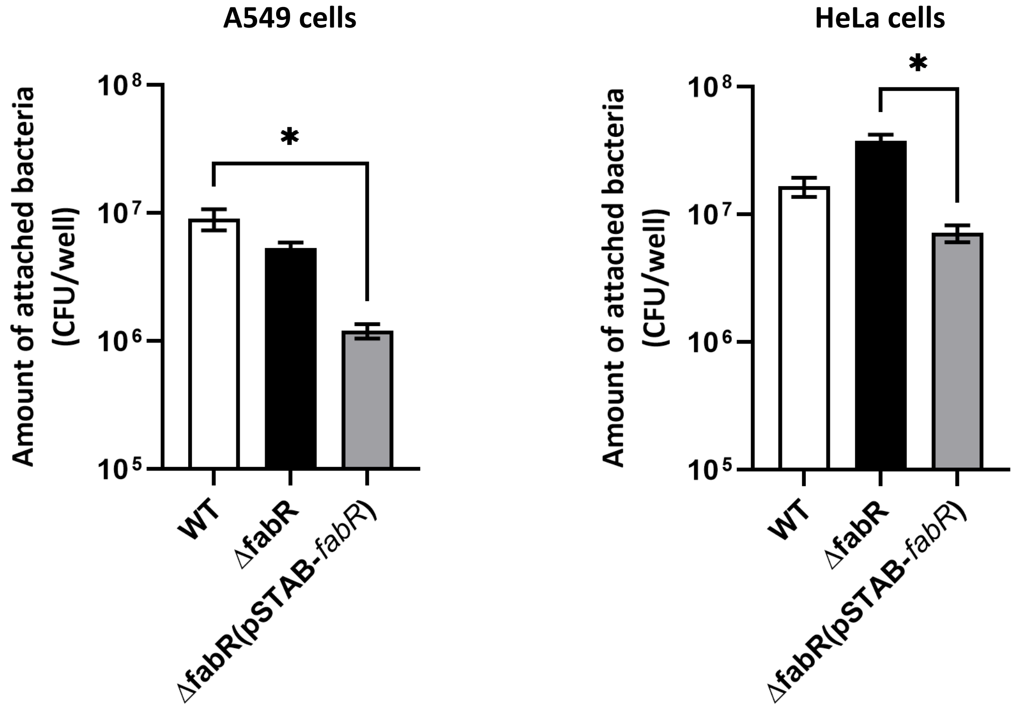

Fig. S2. The deletion of the transcription repressor FabR does not impact the adhesion of K. pneumoniae in eucaryotic cell monolayer. A549 and HeLa epithelial cell monolayers were infected with bacteria such that the multiplicity of infection was 5 bacterium per cell (MOI5). The bacterial adhesion was assessed after 5h of incubation by CFU counting. Results are presented as the number of CFU per well. Each value is the mean of three independent experiments. Data are expressed as means ± SEM (N = 3). Statistical analysis: one-way ANOVA with post-hoc Dunn's test (*, p ≤0.05).
