# Supplementary material for: FabR, a regulator of membrane lipid homeostasis, is involved in Klebsiella pneumoniae biofilm robustness
Source: mBio. 2024 Sep 6;15(10):e01317-24. doi: 10.1128/mbio.01317-24 (PMC11481535; doi:10.1128/mbio.01317-24)
Supplement: Figure S3 — The deletion of fabR is associated to the formation of microcolonies with a rounded structure-shape that grew and were not altered by shear forces over time. [file mbio.01317-24-s0003.pdf]

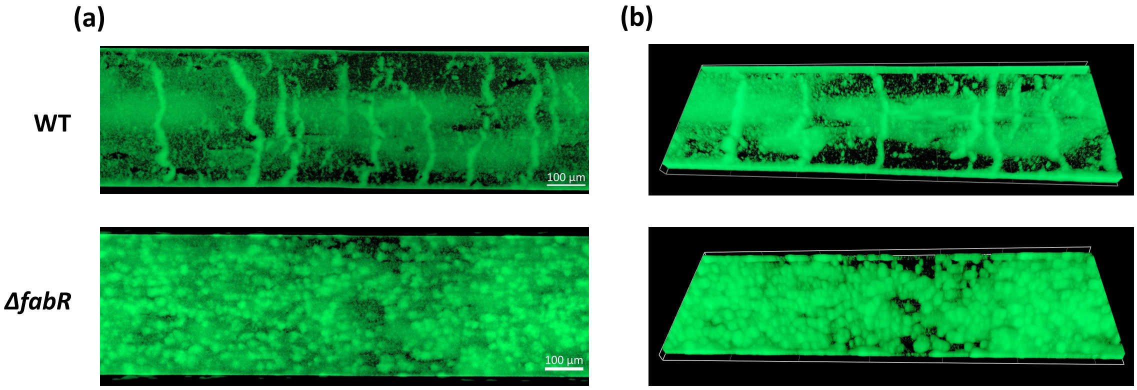

Fig. S3. The deletion of fabR is associated to the formation of microcolonies with a rounded structure-shape that grew and were not altered by shear forces over time. Biofilms formed in the BioFlux^{TM} microfluidic system at 37°C under a shear force of 0.5 dyn/cm² by the GFP-expressing WT and *ΔfabR* strains were observed by epifluorescence microscopy (Axio observer 7, Zeiss) at the magnification of 20 ×. The 2D (a) and 3D (b) image projections of each strain were acquired at T = 12 hours.
