# Supplementary material for: FabR, a regulator of membrane lipid homeostasis, is involved in Klebsiella pneumoniae biofilm robustness
Source: mBio. 2024 Sep 6;15(10):e01317-24. doi: 10.1128/mbio.01317-24 (PMC11481535; doi:10.1128/mbio.01317-24)
Supplement: Figure S4 — Details of the gas chromatography data obtained. [file mbio.01317-24-s0004.pdf]

(a)

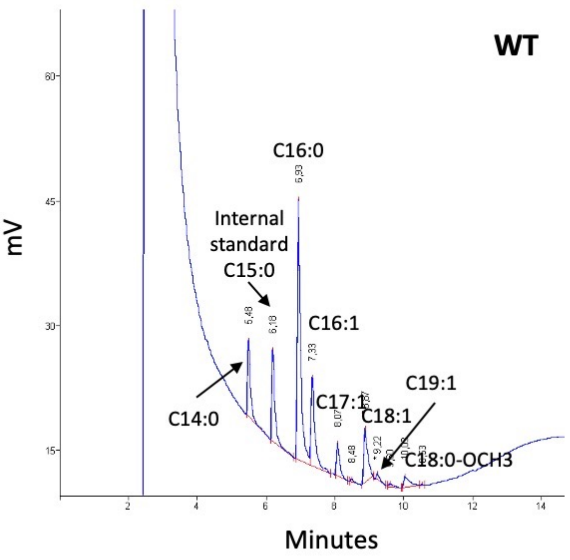

(b)

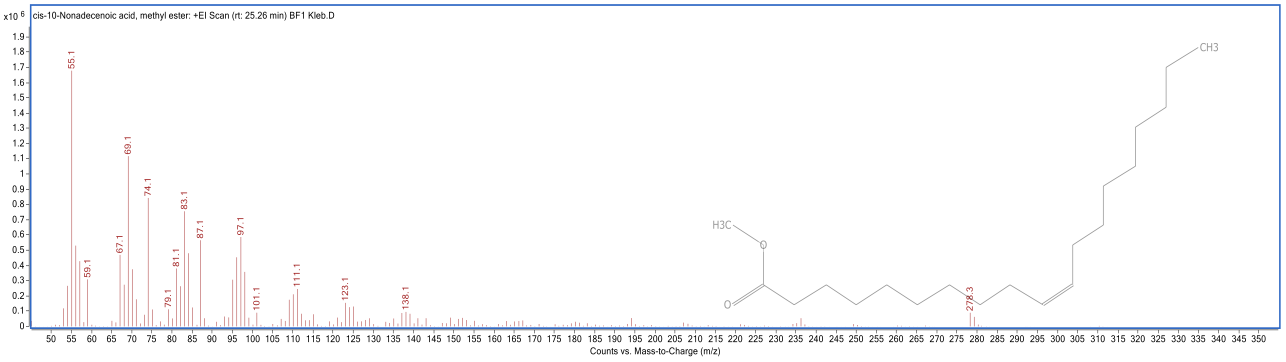

(c)

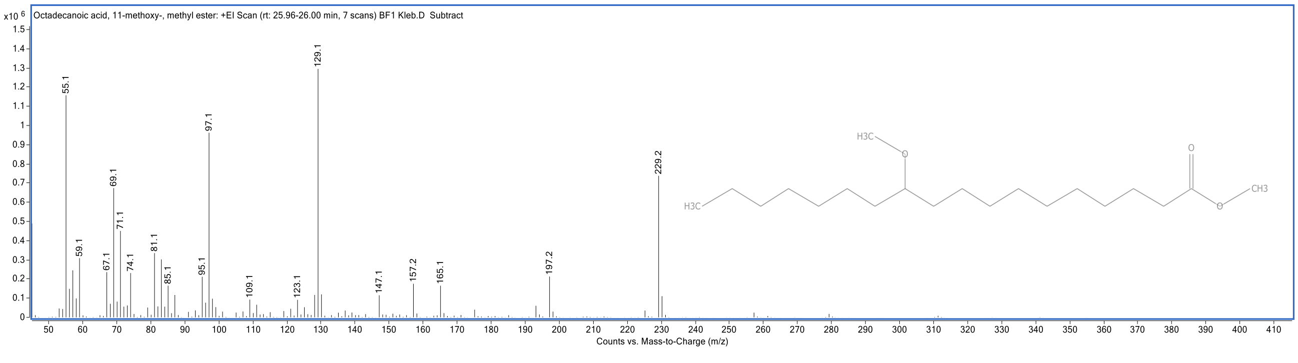

Fig. S4. Details of the gas chromatography data obtained. Chromatogram of the Fatty acid methyl esters (FAMEs) from the biofilm WT with annotation based on commercial standards and GC-MS spectra (a). MS/MS spectrum identified with the NIST library for C19:1 (b) and the MS/MS spectrum identified with the NIST database for C18:0-OCH3 (c).
