# Supplementary material for: FabR, a regulator of membrane lipid homeostasis, is involved in Klebsiella pneumoniae biofilm robustness
Source: mBio. 2024 Sep 6;15(10):e01317-24. doi: 10.1128/mbio.01317-24 (PMC11481535; doi:10.1128/mbio.01317-24)
Supplement: Figure S5 — Identification and quantification of the total fatty acid composition in biofilm cultures were conducted using GC-MS and GC-FID. [file mbio.01317-24-s0005.pdf]

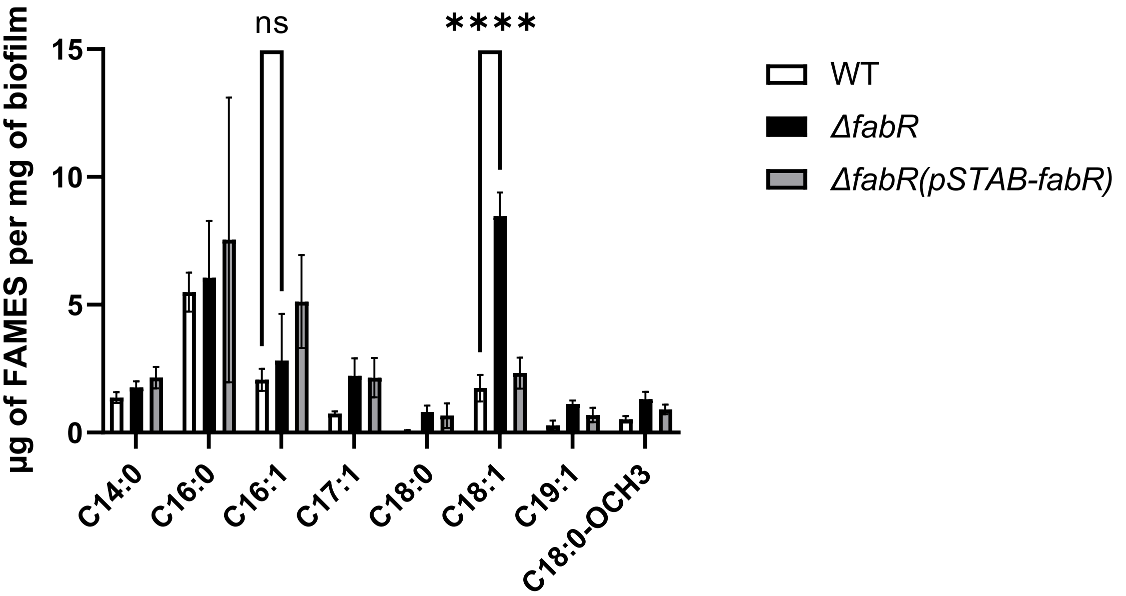

Fig. S5. Identification and quantification of the total fatty acid composition in biofilm cultures were conducted using GC-MS and GC-FID. The fatty acid content in biofilm was analyzed in WT, *ΔfabR*, and Δ*fabR*(pSTAB-*fabR*) biofilm cultures. Fatty acid results are presented in µg of FAMEs per mg of biofilm. The results correspond to at least three or four biologically independent samples. Statistical significances were determined by *post-hoc* Tukey test after two-way analysis of variance (****, $p \leq 0.0001$; ***, $p \leq 0.001$; **, $p \leq 0.01$; *, $p \leq 0.05$).
