# Supplementary material for: FabR, a regulator of membrane lipid homeostasis, is involved in Klebsiella pneumoniae biofilm robustness
Source: mBio. 2024 Sep 6;15(10):e01317-24. doi: 10.1128/mbio.01317-24 (PMC11481535; doi:10.1128/mbio.01317-24)
Supplement: Figure S6 — AFM QI images of K. pneumoniae biofilms showed bacteria embedded in their extracellular matrix of EPS. [file mbio.01317-24-s0006.pdf]

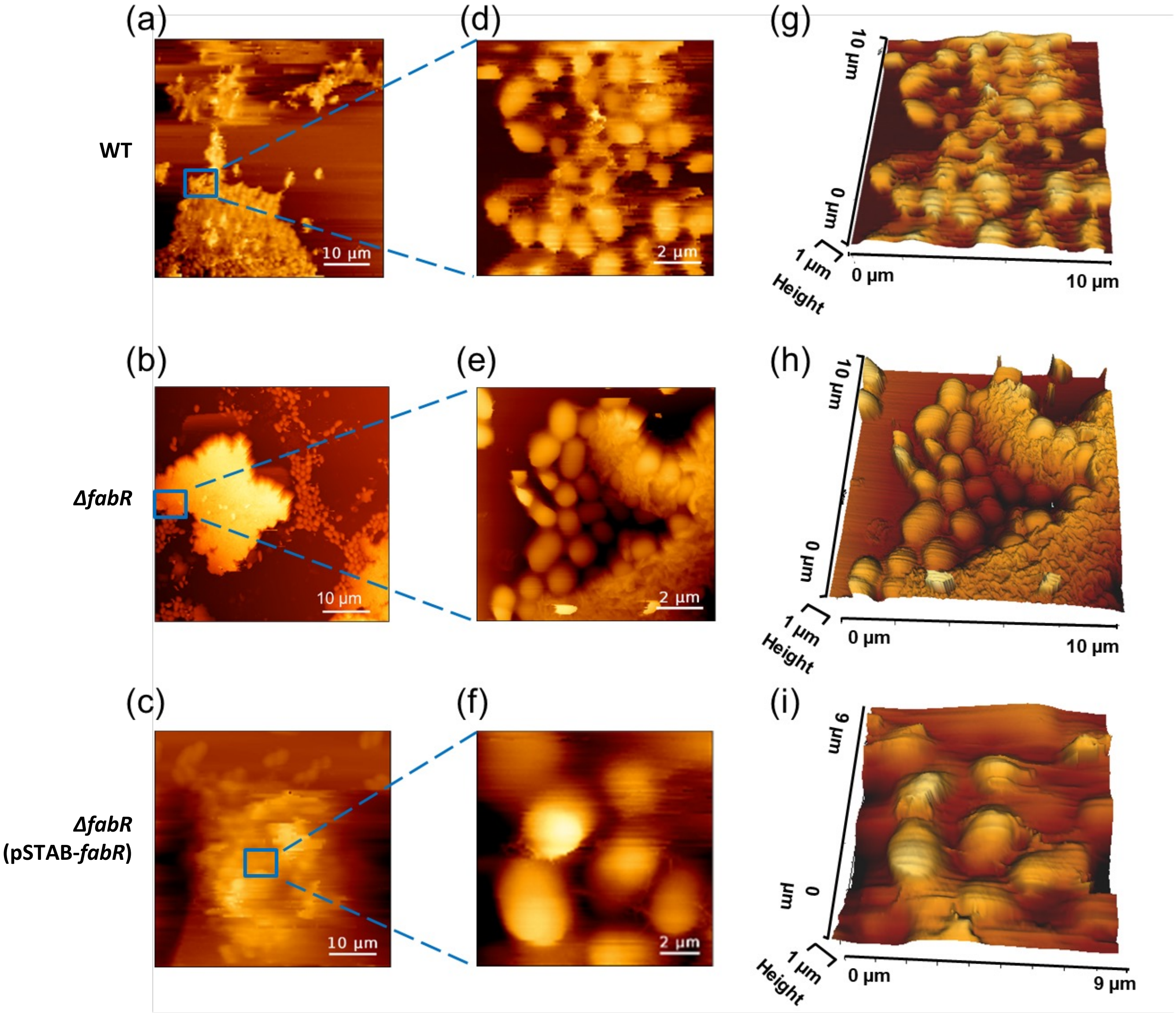


**Fig. S6**. AFM QI™ images of *K. pneumoniae* biofilms showed bacteria embedded in their extracellular matrix of EPS. 2D (a-f) and 3D (g-i) height images were acquired with 5 hour-old biofilm aggregates of WT (a,d,g), *ΔfabR* (b,e,h) and *ΔfabR*(pSTAB-*fabR*) (c,f,i).
