# Supplementary material for: FabR, a regulator of membrane lipid homeostasis, is involved in Klebsiella pneumoniae biofilm robustness
Source: mBio. 2024 Sep 6;15(10):e01317-24. doi: 10.1128/mbio.01317-24 (PMC11481535; doi:10.1128/mbio.01317-24)
Supplement: Figure S7 — The deletion of fabR led to an increase biofilm formation. [file mbio.01317-24-s0007.pdf]

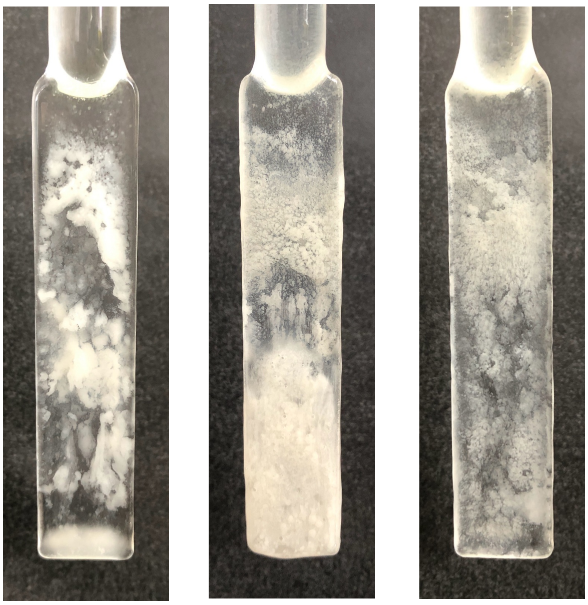

Fig. S7. The deletion of *fabR* led to an increase biofilm formation. Biofilms were formed in a continuous-flow microfermentor on a glass spatula during 24 h. Pictures of the spatula before resuspension are shown.
