# Supplementary material for: FabR, a regulator of membrane lipid homeostasis, is involved in Klebsiella pneumoniae biofilm robustness
Source: mBio. 2024 Sep 6;15(10):e01317-24. doi: 10.1128/mbio.01317-24 (PMC11481535; doi:10.1128/mbio.01317-24)
Supplement: Supplemental text — Supplemental materials and methods. [file mbio.01317-24-s0008.pdf]

Supplemental Materials and Methods

Adhesion to epithelial eucaryotic cells

The cell lines used in this study were maintained in an atmosphere containing 5% CO_2 at 37°C in culture medium supplemented with 10% of heat-inactivated fetal bovine serum (Dominique Dutscher). Human epithelial lung A549 cells (ATCC, CCL-185) and human epithelial cervical HeLa cells (ATCC, CRM-CCL-2) were cultured in Eagle's Minimum Essential Medium and Dulbecco's Modified Eagle medium (Dominique Dutscher, Brumath, France) respectively. Cell monolayers were seeded in 24-well tissue culture plates (Falcon – Corning, New York, USA) at a density of 5.10^5 cells per well on the day before the experiments. The day of experiment, eucaryotic cells were infected with planktonically growing WT, Δ*fabR* and *fabR(*pSTAB-*fabR* bacteria such that the multiplicity of infection was 5 bacterium per cell (MOI_5) in a final volume of 1mL of cell culture media. Plates were centrifugated for 5 min at 500 g, and incubated at 37°C under 5% CO_2. After 3 hours of incubation, cell monolayers were washed two times with PBS, and were incubated for 2 more hours with 1 mL of cell culture media. After incubation, cell monolayers were washed two times with PBS, and lysed with 1% Triton X-100 in PBS. Samples were serially diluted, plated onto LB agar plates, and CFUs were determined.

Analysis of biofilm development in microfluidic system

Biofilm development was analyzed using the BioFlux^{TM} 200 system (Fluxion Biosciences, South San Francisco, CA, USA) microfluidic device with 48-well microplates in which the wells are connected in pairs (between an inlet and an outlet well) via an observation channel (depth=75 µm and width=350 µm). A controlled pneumatic pressure to the top of the inlet well drives the medium fluid through the channels at a user-defined flow rate. The biofilm formation on the cover slip glass bottom of the observation channel is followed in live by microscopy.

To grow biofilm in the BioFlux system, the channels were primed for 5 min with 500 µL of M63B1-0.4 % Glc at 5 dyn/cm². The medium was then aspirated from the output wells and replaced with 100µL of a *K. pneumoniae* overnight culture in M63B1-0.4 % Glc adjusted to OD_{620nm} 0.01 (corresponding to $\sim 10^7$ CFU/mL). The observation channels were seeded by pumping from the output wells to the input wells at 5.0 dyn/cm^2 for 2 seconds. Microplate was then incubated for 15 min at 37°C without shear (adhesion step) inside the temperature-controlled chamber of the microscope. After incubation, the biofilm formation was monitored

at 37°C under a shear force of 0.5 dyn/cm² (corresponding to a flow rate of 63 µL/h) using an inverted epifluorescence microscope Axio observer 7 (Zeiss) at the magnification of 20x. Images were acquired every 1 hour for 19 hours and analyzed with Zeiss Zen 2 (blue edition) software. The mechanical strength of biofilms was assessed after 19 hours of culture by gradually increasing the shear force from 0.5 to 10 dyn/cm².

RNA extraction and reverse transcription-quantitative PCR assay

Planktonic bacteria were recovered from culture in M63B1-0.4% Glc and biofilms were recovered from the glass slide of the flow-cell after a 24 hour-incubation period (see materials and methods). For both planktonic and biofilm samples, an equivalent of 3 units of OD_{620nm} were pelleted by centrifugation at 6 000 g for 5 min at 4°C, and pellets were resuspended in 1 mL of PBS and 2 mL of RNAprotect bacteria reagent (Qiagen) to avoid transcriptional changes and RNA degradation. Bacteria sampled were stored at -80 °C until RNA extraction.

Total RNA was extracted according to the method described by Toledo-Arana *et al*. (1). Briefly, bacteria were mechanically lysed with the PreCellys 24 system (Bertin Technologies, Montigny le Bretonneux, France) at speed of 6 500 rpm for two consecutive cycles of 30 s. After acid phenol (Thermo Fisher Scientific) and TRIzol® (Thermo Fisher Scientific) extraction, total RNA was precipitated with isopropanol and treated with 10 units of TURBO DNase (Thermo Fisher Scientific). After a second phenol-chloroform extraction and ethanol precipitation, RNA pellets were suspended in DEPC-treated water. RNA concentrations were quantified with the Qubit system (Thermo Fisher Scientific). The absence of DNA contamination was verified by qPCRs performed with primer pair RT-proC-Fw/RT-proC-Rv (**Supplemental Table**) and the SsoAdvanced SYBR® Green Supermix (Bio-Rad, Hercules, California, USA) according to the manufacturer's recommendations.

Reverse transcription was performed with 500 ng of total RNA using the iScript cDNA Synthesis kit (Bio-Rad) according to the manufacturer's recommendations. qPCRs were carried out in the CFX96 Real Time System (Bio-Rad) with the SsoAdvanced SYBR® Green Supermix (Bio-Rad) in 10 µL total volume per well with 2 µL of 10X diluted cDNA. Primers were designed on the basis of *K. pneumoniae* CH1157 genome sequence information and are listed in Supplemental Table. The gene expression levels were normalized relative to the expression level of the *proC and rpoD* housekeeping genes (2). The relative expression of the genes *fabA*, *fabB* and that

encoding the putative fatty acid desaturase DesA_FADS-like were determined with CFX Manager software (Bio-Rad).

1. Toledo-Arana A, Dussurget O, Nikitas G, Sesto N, Guet-Revillet H, Balestrino D, Loh E, Gripenland J, Tiensuu T, Vaitkevicius K, Barthelemy M, Vergassola M, Nahori MA, Soubigou G, Regnault B, Coppee JY, Lecuit M, Johansson J, Cossart P. 2009. The *Listeria* transcriptional landscape from saprophytism to virulence. Nature 459:950–6.
2. Gomes AÉI, Stuchi LP, Siqueira NMG, Henrique JB, Vicentini R, Ribeiro ML, Darrieux M, Ferraz LFC. 2018. Selection and validation of reference genes for gene expression studies in *Klebsiella pneumoniae* using Reverse Transcription Quantitative real-time PCR. Sci Rep 8:9001.
