# Supplementary material for: FabR, a regulator of membrane lipid homeostasis, is involved in Klebsiella pneumoniae biofilm robustness
Source: mBio. 2024 Sep 6;15(10):e01317-24. doi: 10.1128/mbio.01317-24 (PMC11481535; doi:10.1128/mbio.01317-24)
Supplement: TABLE S1 — Primers used in this study. [file mbio.01317-24-s0009.pdf]

Table S1. Primers used in this study

Primer name	Primer sequence (5'- 3')	Use
FabR-KmFRT-Fw	*ACCGATTAGTTTTAATCTCTGGTATAGTGCCAGCAGGGCTATTGGAAGGA TTCATACATC*GTCTTGAGCGATTGTGTAGG	Amplification of kanamycin resistance encoding gene flanked by FRT sites to create the *fabR* isogenic mutant
FabR-KmFRT-Rv	*CAGCGCCAGCAGCAGCGTACCTTTATCTTGAACTGAGTGTTTCATTGCTC GTCCTTCACG*TGGTCCATATGAATATCCTCC	
Verif-FabR-Fw<br>Verif-FabR-Rv	ACACGATGATAACATTCGCTG<br>ACGGAACTATCGAACTGAAC	Verification of *fabR* deletion in *K. pneumoniae* chromosome
Gibson-pSTAB-Fw<br>Gibson-pSTAB-Rv	GGTACCCCCGTACAATGCC<br>GAATTCGGTCAGTGCGTCCT	Amplification of pSTAB plasmid
fabR-Gib-pSTAB-Fw<br>fabR-Gib-pSTAB-Rv	*TCAGCAGGACGCACTGACCGAATTC*CACGATGATAACATTCGCTGC<br>*CTTGCTGGCATTGTACGGGGGTACC*AACTGAGTGTTTCATTGCTCG	Amplification of *fabR* for cloning in pSTAB plasmid
pSTAB-Fw<br>pSTAB-Rv	GCGGATAACAAGATACTGAGC<br>CTGCACTGAAGCGCCTTTAT	Verification of the presence of insert in pSTAB plasmid
RT-fabA-Fw<br>RT-fabA-Rv	TGATGGACCGCGTCATCAAA<br>CCGAAGAACCACAGGTCAGG	Quantification of *fabA* expression by qPCR
RT-fabB-Fw<br>RT-fabB-Rv	ACGTATGGGGCAACGTCAAA<br>GATGGAGGCGTCGCTCATAA	Quantification of *fabB* expression by qPCR
RT-desA-Fw<br>RT-desA-Rv	TTAACCAGCTGTTGGGGACG<br>GTCAGCAGATGGTTGCGATG	Quantification of *desA* expression by qPCR
RT-fabR-Fw<br>RT-fabR-Rv	GGCATTCAGCCAACTTAGCG<br>GACGATAAAAGGACGTCGGC	Quantification of *fabR* expression by qPCR
RT-yqfA-Fw<br>RT-yqfA-Rv	CCACTGCGCCATCTATTTGC<br>TGACGATCATCAGCCCCTTC	Quantification of *yqfA* expression by qPCR
RT-proC-Fw<br>RT-proC-Rv	GATTGCCGATATCGTCTTCG<br>GAGACCACCAGCGACTCTTT	Quantification of *proC* expression by qPCR
RT-recA-Fw<br>RT-recA-Rv	TTAAACAGGCCGAATTCCAG<br>CCGCTTTCTCAATCAGCTTC	Quantification of *recA* expression by qPCR
